# Molecular Insights into Adhesion at Interface of Geopolymer Binder and Cement Mortar

**DOI:** 10.3390/ijms25158374

**Published:** 2024-07-31

**Authors:** Anton S. Kasprzhitskii, Alexander A. Kruglikov

**Affiliations:** Laboratory of Fuel Energy Waste Recycling, Platov South-Russian State Polytechnic University (NPI), Prosveshcheniya St., 132, Rostov Region, Novocherkassk 346428, Russia; aleksan.kruglikov@yandex.ru

**Keywords:** geopolymer, oligomer, cement mortar, concrete, repair materials, interaction mechanism, density functional theory, Monte Carlo simulations

## Abstract

The degradation of concrete and reinforced concrete structures is a significant technical and economic challenge, requiring continuous repair and rehabilitation throughout their service life. Geopolymers (GPs), known for their high mechanical strength, low shrinkage, and durability, are being increasingly considered as alternatives to traditional repair materials. However, there is currently a lack of understanding regarding the interface bond properties between new geopolymer layers and old concrete substrates. In this paper, using advanced computational techniques, including quantum mechanical calculations and stochastic modeling, we explored the adsorption behavior and interaction mechanism of aluminosilicate oligomers with different Si/Al ratios forming the geopolymer gel structure and calcium silicate hydrate as the substrate at the interface bond region. We analyzed the electron density distributions of the highest occupied and lowest unoccupied molecular orbitals, examined the reactivity indices based on electron density functional theory, performed Mulliken charge population analysis, and evaluated global reactivity descriptors for the considered oligomers. The results elucidate the mechanisms of local and global reactivity of the oligomers, the equilibrium low-energy configurations of the oligomer structures adsorbed on the surface of C-(A)-S-H(I) (100), and their adsorption energies. These findings contribute to a better understanding of the adhesion properties of geopolymers and their potential as effective repair materials.

## 1. Introduction

Degradation of concrete and reinforced concrete structures is a serious technical and economic problem, requiring timely and effective preventive measures, as well as repair and rehabilitation throughout their service life [1,2]. Repair materials (RMs) play an important role in extending the service life and durability of engineering infrastructure, providing a reduction in operating costs and improving safety [3,4,5]. Such materials are commonly used in engineering practice to repair large cracks, fill hollows or cavities, and coat surfaces for repairing deteriorated and damaged concrete structures. In the context of concrete repair, cement mortar plays a crucial role as it forms the interface between the existing concrete substrate and new repair materials. The adhesion and compatibility of repair materials with the cement mortar substrate are critical factors determining the effectiveness and longevity of concrete repairs. This study focuses on the interaction between geopolymer-based repair materials and the cement mortar substrate, specifically investigating the molecular-level processes at this interface.

Recently, geopolymers (GPs) produced by low-temperature alkaline activation of aluminosilicate materials, including industrial solid wastes and by-products (red mud [6], granulated blast furnace slag [7], fly ash [8], electric furnace nickel slag [9], and binary and ternary waste blends [10,11,12]), have been increasingly considered as an alternative to conventional repair materials. Geopolymers are characterized by their relatively high mechanical strength [13], low shrinkage [14], and durability [15], making them potentially attractive as a base for repair mixtures. The presence of reactive phases with relatively low Al_2_O_3_ content but large amounts of reactive SiO_2_ and CaO in some waste materials suitable for geopolymers promotes the formation of N(C)-A-S-H type gel phases, thus reducing the shrinkage of the material upon drying [16,17], which is critical for repair materials.

In order to perform their functions effectively, repair mixtures must have good adhesion to the substrate surface, sufficient flexibility and strength to withstand mechanical stresses, and the ability to endure internal stress changes caused by volume changes and chemical and electrochemical factors [18]. Due to differences in elastic properties, thermal coefficients, and shrinkage between the substrate and overlay, the interface bond region is usually the weakest link in the system [19,20]. This makes it difficult to form a tightly bonded interfacial layer between the old concrete substrate and the new repair material, which, over time, leads to crack formation, reducing the filling efficiency and load transfer capability. In some cases, the repair material may delaminate from the substrate, making repairs ineffective. The addition of granulated blast furnace slag to metakaolin-based geopolymer is an option to improve the interfacial chemical bonding force due to the secondary hydration reaction of GP, as observed by Fan et al. [7]. With increasing calcium content, more C-S-H and/or C-A-S-H at the interface is formed, promoting better adhesion [21]. In turn, an increase in the Si/Al ratio in geopolymer mixes can lead to an increase in substrate-to-overlay interfacial bond strength in concrete repairs, as reported by Asayesh et al. [22].

Although more and more papers have reported on the prospects of using geopolymer mortars as sustainable repair materials [23], there is currently a gap in understanding the interface bond properties at the site of a new geopolymer layer on the old concrete substrate. The influence of material factors controlling adhesive failure, in particular the chemical composition of the geopolymer binder, remains understudied. A better understanding of interfacial bonding mechanisms is important to assess the potential of geopolymers as repair materials.

In the present work, utilizing quantum mechanical approaches and stochastic simulations, we examined the adsorption characteristics and bonding mechanisms of aluminosilicate oligomers with varying Si/Al ratios. These oligomers, which form the geopolymer gel structure, were studied in relation to calcium silicate hydrate, which served as a substrate at the interface bond region. We analyzed the spatial distribution of electron density for frontier molecular orbitals, evaluated reactivity indicators derived from electron density functional theory, conducted Mulliken charge distribution analysis, and calculated global reactivity parameters for the oligomers under consideration. We determined the stable configurations of oligomers adsorbed on the C-(A)-S-H(I) (100) surface that minimize energy, and computed their respective adsorption energies. This approach allows us to gain insights into the molecular-level interactions between geopolymer-based repair materials and the cement mortar substrate, which is crucial for understanding and improving the adhesion in concrete repair applications.

## 2. Results and Discussion

### 2.1. Local Reactivity of Aluminosilicate Oligomers

**Figure 1:**
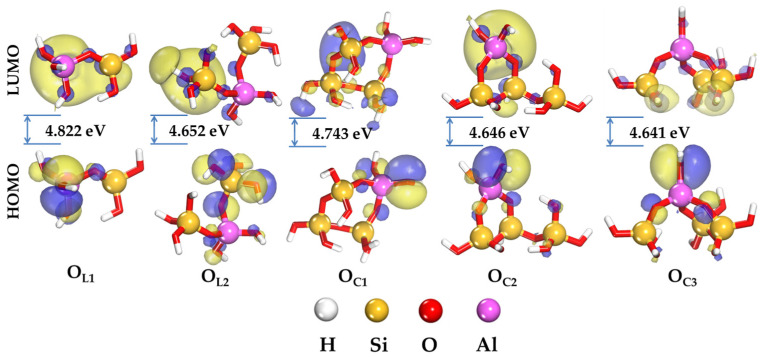
HOMO and LUMO isosurface densities of O_L_ and O_C_ oligomers in the gas phase (value for isosurface: 0.03 e∙Å^−2^).

The study of the local reactivity of linear (O_L_)- and ring (O_C_)-type aluminosilicate oligomers is crucial for understanding their interaction with the cement mortar substrate at the molecular level. This analysis is performed for the equilibrium structures of the oligomers, simulating their state when in contact with the cement mortar surface during the application of geopolymer repair materials. The assessment of reactivity for these multi-atomic complexes is founded on Fukui’s frontier orbital theory, which is particularly relevant for predicting chemical interactions at the geopolymer–cement mortar interface. It is dependent on the energetic positions of the frontier molecular orbitals, namely, the highest occupied molecular orbital (HOMO) and the lowest unoccupied molecular orbital (LUMO), which govern the nature of donor–acceptor interactions between the oligomers and the cement mortar surface [24]. The maximum value of E_HOMO_ corresponds to the highest propensity of multi-atomic systems to release electrons. Conversely, a low E_LUMO_ value indicates an enhanced capability to accept electrons into the unoccupied electronic levels of polyatomic systems [25]. These energetic criteria are commonly employed to assess their interaction with atomic surfaces [26]. In this context, an increase in the E_HOMO_ value coupled with a decrease in E_LUMO_ is expected to yield the most favorable adhesion effect with the surface. A crucial energetic parameter characterizing the activity of a multi-atomic system is the energy gap (ΔE = E_LUMO_ − E_HOMO_) between E_LUMO_ and E_HOMO_. This parameter allows us to elucidate the reactivity of oligomers during adsorption onto the atomic surface [27], simulating their behavior when applied to cement mortar. A reduction in ΔE signifies an enhancement in the reactivity of the oligomer, which promotes improved adhesion to the cement mortar substrate. The computational results presented in Figure 1 demonstrate that for linear aluminosilicate oligomers, ΔE ranges from 4.822 to 4.652 eV for O_L1_ and O_L2_, respectively. Thus, the O_L_ reactivity increases with the growing number of silicon-containing octahedra. This implies an increase in atomic surface affinity for the oligomers with increasing aluminosilicate framework Si/Al ratio. This trend is consistent with observations reported for other polyatomic systems [28,29].

The presence of ring structures in the aluminosilicate framework of O_C_ oligomers leads to an increase in their reactivity. The energy gap values shown in Figure 1 for these oligomers decrease from 4.743 to 4.641 eV for O_C1_ and O_C3_, respectively. The E_HOMO_ value for the linear oligomers O_L1_ and O_L2_ changes from −6.310 eV to −6.284 eV, respectively. A larger effect is observed for oligomers with O_C_ ring structures, for which the E_HOMO_ value increases by 0.18 eV when going to O_C3_. The increase in Si/Al changes the E_LUMO_ level by 0.14 eV and 0.30 eV for linear and ring-shaped oligomers, respectively. This observation suggests an enhanced electron-accepting capacity for oligomers with increasing Si/Al ratio when alumino- and silica-containing tetrahedrons are integrated into a common framework.

Figure 1 illustrates the spatial arrangement of frontier molecular orbitals for oligomers with linear and ring forms at an isosurface value of 0.03. The localization of electron density associated with the HOMO and LUMO frontier orbitals on specific atomic groups indicates reactive sites involved in donor–acceptor interactions with the mineral surface [25]. As can be seen in Figure 1, the spatial distribution of electron density for the frontier orbitals is influenced by the structure of the aluminosilicate framework. For the linear oligomer O_L1_, the electron density is distributed throughout the framework, whereas for the ring oligomers O_C_, it is concentrated on specific functional molecular groups. In this case, due to OH groups, which can establish additional chemical bonds with the surface atoms of calcium silicate hydrate, a parallel interaction of O_C_ with the mineral surface can be postulated. These findings are corroborated by the adsorption configurations obtained from stochastic simulations presented in the subsequent section of this work. For the linear form of O_L_ oligomers, the electron-donor and electron-acceptor sites are primarily associated with the OH groups of the silicic-oxygen or alumino-oxygen tetrahedra. In contrast, for the ring forms of O_C_ oligomers, the electron-donor sites are predominantly located in the vicinity of the aluminosilicate tetrahedron, while the electron-acceptor sites are mainly found in the region of the silica/silicate tetrahedron. The overall comparative analysis suggests that O_C_ ring oligomers have a greater propensity for electron exchange with multiple sites on the cement mortar surface, potentially facilitating the formation of stronger chemical bonds at the geopolymer–cement mortar interface. This implies their efficient adsorption onto the surface of the cement mortar substrate, potentially resulting in the formation of a stable and strongly adhered geopolymer repair layer.

Further insights into the local reactivity of aluminosilicate oligomers can be gained through the application of Fukui’s concept [24]. The Fukui function, $f\left(r\right)$, is mathematically defined as the first derivative of the electron density $\rho \left(r\right)$ with respect to the number of electrons *N*. This function enables the identification of atoms within the molecule that are prone to nucleophilic and electrophilic reactions. Employing the finite difference approximation and utilizing the charge of the atom in its neutral state $q\left(N\right)$ and its cationic state $q\left(N-1\right)$, the Fukui functions can be computed as follows [29]:

$$f^{+}=q\left(N+1\right)-q\left(N\right)\;\mathrm{for}\;\mathrm{nucleophilic}\;\mathrm{attack};\;f^{-}=q\left(N\right)-q\left(N-1\right)\;\mathrm{for}\;\mathrm{electrophilic}\;\mathrm{attack}$$

**Figure 2:**
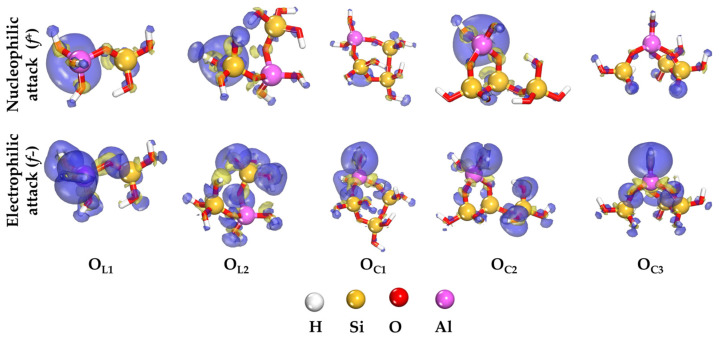
Spatial distribution of reactivity indicators derived from electron density functional theory for aluminosilicate oligomers in the gas phase (isosurface value: 0.01).

Figure 2 depicts the spatial distribution of $f^{+}\;\;$ and $f^{-}\;\;$ for oligomers in linear and ring conformations at an isosurface value of 0.01. For oligomers in linear form, there is a notable prevalence of sites susceptible to electrophilic attack compared to those prone to nucleophilic attack. In the case of ring-form oligomers, the nucleophilic attack centers are primarily localized on OH groups of organosilicon tetrahedra, while the regions vulnerable to electrophilic attack are predominantly associated with OH groups of alumina tetrahedra.

The comprehensive analysis reveals a proliferation of reactive sites within the framework structure as the Si/Al ratio increases, attributed to the emergence of additional OH groups. The ring conformation of the oligomer exhibits enhanced reactivity compared to its linear counterpart. The observed increase in potential reaction sites capable of interacting with the mineral surface suggests an improved likelihood of achieving effective adhesion.

**Figure 3:**
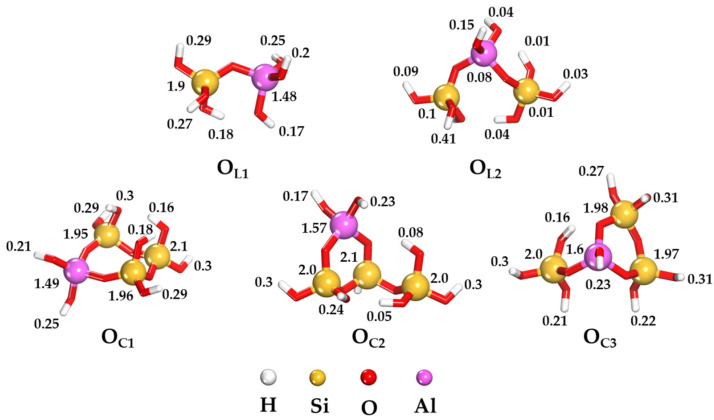
Mulliken charges for aluminosilicate oligomers in the gas phase.

To further elucidate the atomic-level adsorption mechanisms of the oligomers, we conducted a Mulliken population analysis on the framework structure [30]. It is well-established that atoms with more negative charge exhibit a higher propensity for surface adsorption through donor–acceptor interactions. The outcomes of this analysis for both linear and ring conformations of the oligomers are summarized in Figure 3. The graphical representation clearly demonstrates that the ring structure possesses a higher concentration of negative charge on its heteroatoms compared to the linear configuration. This observation lends support to the enhanced reactivity of the ring forms of O_C1_, O_C2_, and O_C3_ oligomers.

### 2.2. Global Reactivity of Aluminosilicate Oligomers

**Figure 4:**
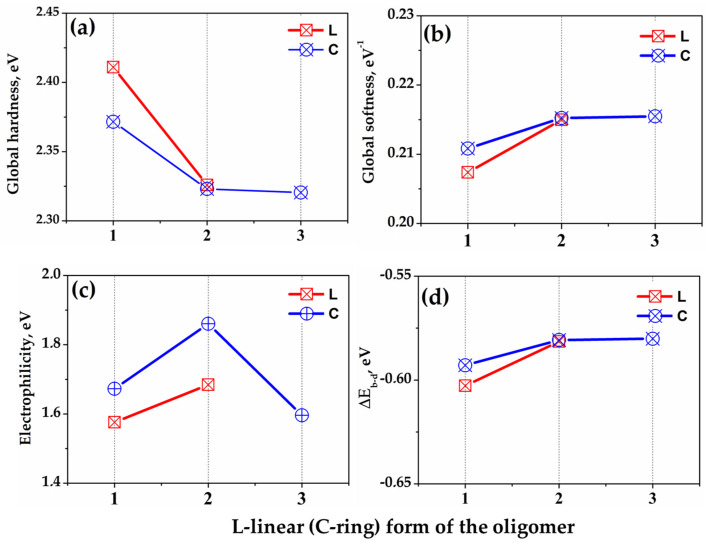
Descriptors of global reactivity of aluminosilicate oligomers in the gas phase. (**a**) global hardness, (**b**) global softness, (**c**) electrophilicity (**d**) ΔE_b-d′_, L- linear form of the oligomer, C- ring form of the oligomer.

Global reactivity indicators serve as crucial parameters for predicting the adhesive properties of multi-atomic systems, which is particularly relevant when considering the interaction between geopolymer repair materials and cement mortar substrates. The quantification of these descriptors was carried out in accordance with the principles outlined in Koopmans’ theorem [31], utilizing the energetic values associated with the HOMO and LUMO. The outcomes of these calculations for O_L_ and O_C_ oligomers in both linear and ring conformations, which represent different structural components of geopolymers, are presented in Figure 4. These results provide insights into how different geopolymer structures might interact with the cement mortar surface at a molecular level.

The assessment of global hardness (softness) is illustrated in Figure 4a,b. These parameters are evaluated in alignment with the hard and soft acids and bases (HSABs) principle [32]. This principle posits that compounds preferentially react with systems exhibiting comparable hardness (softness) characteristics. In the context of concrete repair, this suggests that geopolymer components with softness values closer to those of the cement mortar surface are likely to form stronger bonds. The strength of interaction is expected to increase as the hardness (softness) of the reactants decreases [33]. Consequently, the interaction with the mineral surface is enhanced for softer polyatomic systems (characterized by lower hardness and higher softness values) compared to harder polyatomic systems (exhibiting higher hardness and lower softness values). The data presented in Figure 4a,b indicate an enhancement in global softness associated with the formation of the O_C_ ring structure (O_C1_, O_C2_, and O_C3_). Notably higher global softness values are observed for the ring-form oligomers relative to their linear counterparts. These findings suggest that the ring configuration of the oligomer exhibits superior reactivity compared to the linear form.

The concept of electrophilicity provides a measure of a polyatomic system’s capacity to accept electrons. In this context, a higher electrophilicity value corresponds to an increased propensity for electron acceptance, thus indicating stronger electrophilic behavior, and vice versa. The results depicted in Figure 4c demonstrate an intensification of electron-accepting processes with increasing Si/Al ratio for linear oligomers. Conversely, for ring-shaped oligomers, an increase in Si/Al ratio is associated with enhanced electron-donating capabilities.

The exchange of charge between the oligomer and the mineral surface, characteristic of donor–acceptor processes, can be evaluated through the analysis of energy variations [26]. The energy change calculations, as shown in Figure 4d, yield negative values for all oligomers. This observation indicates that both the forward donation and reverse donation processes are energetically favorable. The magnitude of this energy parameter increases with rising Si/Al ratio, suggesting enhanced adsorption of oligomers at higher Si/Al ratios. It is noteworthy that this trend is more pronounced for ring-form oligomers compared to their linear counterparts.

### 2.3. Adsorption Behavior of Aluminosilicate Oligomers

The adhesion efficacy of O_L_ and O_C_ oligomers was investigated in relation to calcium silicate hydrate, a widely used model for the primary binding phase in concrete. This approach allows us to simulate and understand the interactions between geopolymer repair materials and the cement mortar substrate at a molecular level. This investigation was conducted through a comprehensive examination of the adsorption characteristics derived from stochastic modeling techniques. We evaluated the energetically favorable adsorption configurations of the oligomers on the C-S-H surface and quantified their corresponding adsorption energies. The energy released during the adsorption process (E_ads_) when the relaxed adsorbate components interact with the substrate was determined using the following equation:

E_ads_ = E_Total_ − (E_Adsorbate_ + E_Substrate_)

where E_ads_ represents the energy of the substrate–adsorbate system, while E_Adsorbate_ and E_Substrate_ denote the energies of the isolated oligomer and mineral surface, respectively.

**Figure 5:**
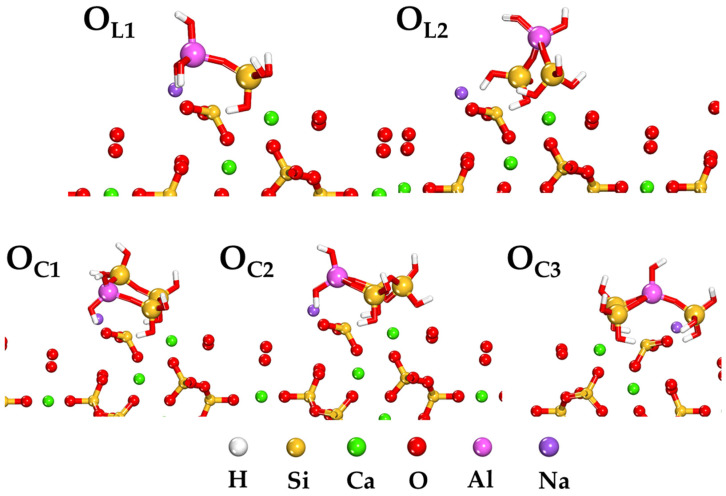
Energetically favorable configurations determined through stochastic simulations for the adsorption of O_L_ and O_C_ oligomers on the C-(A)-S-H(I) surface (100).

The computationally derived low-energy structural arrangements of O_L_ and O_C_ oligomers adsorbed onto the C-(A)-S-H(I) (100) surface are illustrated in Figure 5. The simulations reveal that all oligomers adopt a parallel orientation with respect to the mineral surface upon adsorption. This behavior is consistent across both linear and ring conformations of the oligomers. The observed spatial arrangement relative to the C-(A)-S-H(I) (100) surface can be attributed to the distribution of HOMO and LUMO electron densities within the oligomers. This suggests that the adsorption process involves bidirectional electron transfer between the oligomers and the investigated mineral surface. The parallel alignment indicates enhanced adhesive capabilities of the oligomers and promotes maximal coverage of the mineral surface.

The predominant types of adsorbate–substrate interactions can be inferred through an analysis of bond lengths. Typical intermolecular distances associated with van der Waals interactions, mineral complexation, and hydrogen bonding fall within the ranges of 5–10 Å, 2–3 Å, and 2–3.5 Å, respectively. Table 1 presents the minimum distances between the surface metal atoms and the nearest aluminum and silicon atoms of the adsorbed oligomers in their equilibrium configurations. The majority of these interatomic distances are less than 3.5 Å, indicating the formation of robust chemical bonds between the chemisorbed oligomers and the mineral surface. Exceptions to this trend are observed in the Al-O bond lengths of the linear oligomer O_L2_ and the Si-O bond lengths in the ring-form oligomer O_C1_. The data in Table 1 demonstrates that the adsorption process results in a progressive decrease in the analyzed bond lengths, following the sequence: O_C3_ > O_C2_ > O_C1_. This trend suggests that ring-shaped oligomers exhibit stronger adsorption characteristics on the investigated mineral surface compared to their linear counterparts.

**Figure 6:**
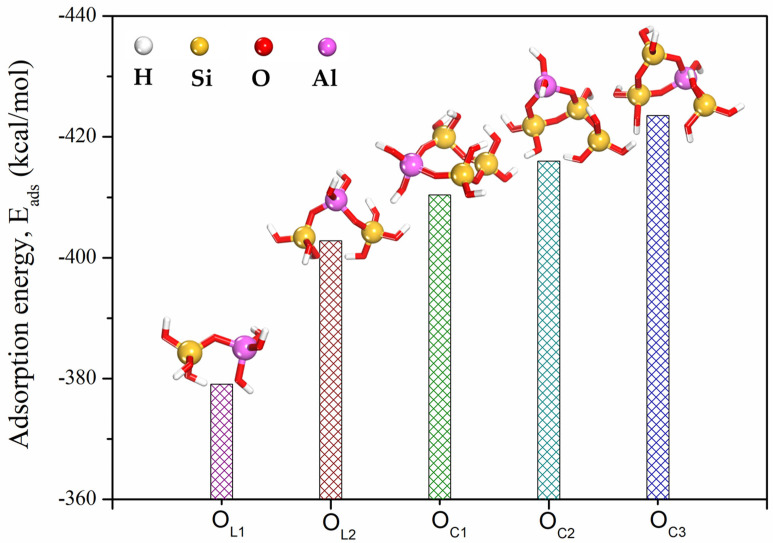
Adsorption energies obtained from stochastic simulations for the adsorption of O_L_ and O_C_ oligomers onto the C-(A)-S-H(I) surface (100) in the gas phase.

Figure 6 illustrates that an increase in the Si/Al ratio of the aluminosilicate framework correlates with a significant reduction in Eads. The observed high negative values of adsorption energy for the oligomers are indicative of their enhanced adsorption capacity and, by extension, their adhesion efficiency. These computational results align well with existing experimental studies [34,35,36,37]. Research by [34] established that the Si/Al ratio in geopolymers plays a crucial role in determining both compressive strength and adhesion strength, which are essential properties for the material’s effectiveness as a coating. While a higher Si/Al ratio generally enhances the compressive strength of the geopolymer, its impact on adhesion strength can vary depending on factors such as substrate type and surface roughness.

A study conducted by [35] demonstrated that elevated Si/Al ratios strengthen the bonding between the geopolymer binder and the substrate, resulting in improved durability and effectiveness in high-temperature and aggressive environments. The research showed that an appropriate Si/Al ratio contributes to the chemical stability of the geopolymer matrix, which in turn influences the longevity and adhesion strength of coatings. The formation of stable chemical bonds leads to increased adhesion [36].

Furthermore, a higher Si/Al ratio has been associated with the development of a denser and more cohesive microstructure, which can enhance the adhesion strength of geopolymer coatings by reducing porosity and improving interfacial bonding [36]. In a study by [37], it was revealed that the Si/Al ratio significantly influences the adhesion strength of geopolymer coatings. Higher ratios generally lead to improved adhesion, attributed to the increased silicon dioxide content, which enhances the geopolymerization process and results in a more condensed structure. Notably, the maximum adhesion strength was observed at a Si/Al ratio of 3, which aligns closely with the computational results obtained in this work. The highest negative adsorption energy is associated with O_C3_ ring-shaped oligomers (Si/Al = 3), indicating their superior potential for adhesion. This finding is in agreement with the quantum chemical calculations presented in the preceding sections of this study.

## 3. Materials and Methods

**Figure 7:**
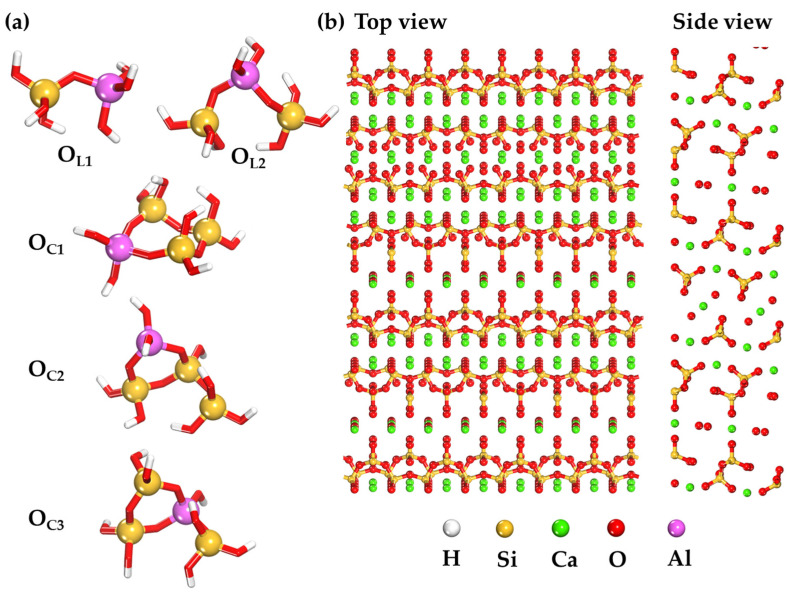
(**a**) O_L_ and O_S_ oligomers defining the structure of N-A-S-H aluminosilicate gel; (**b**) surface model of the C-(A)-S-H(I) surface (100).

A cross-linked three-dimensional N-A-S-H structure is formed during the geopolymerization of oligomers containing silicon and aluminum [38]. These oligomers (monomers), containing Si and Al, are structurally represented as oxygen tetrahedral complexes [39]. During bond formation between monomers, the Loewenstein principle is observed, meaning that two Al tetrahedra cannot be linked by a single oxygen bridge. Therefore, each Al tetrahedron is always connected to four Si tetrahedra [40]. The formation of dimers and trimers, as a reaction between monomers containing Si and Al(OH)_4_^−^, and their existence in isolated form, is consistent with nuclear magnetic resonance (NMR) experimental data [41,42,43]. Increasing the pH of the alkaline solution further facilitates the reaction between monomers, making the environment more favorable for the formation of complex structures such as dimers and trimers at the initial stage of geopolymerization, as discussed in studies [38,39,44]. However, it is acknowledged that aluminum coordination can vary in real-world silicate-containing systems. Recent literature, such as [45], has found that Al coordination varies and Al-IV facilitates oligomerization in neutral conditions. Models may not fully account for the effects of various reaction conditions, such as temperature, pH, and concentration, which can significantly influence the behavior of aluminosilicate oligomers [46]. The transition from oligomers to larger structures, including nucleation and growth processes, is difficult to capture accurately in current models [46,47]. This highlights the complexity of real-world systems compared to the pure, ordered models used in this study. In this work, five oligomers (Figure 7a) with different spatial configurations and Si/Al ratios (1 to 3) were investigated. Linear-type aluminosilicate oligomers with Si/Al ratios from 1 to 2 (O_L1_ and O_L2_) were considered, as well as aluminosilicate oligomers in the form of O_C1_, O_C2_, and O_C3_, presented with a Si/Al ratio of 3, as shown in Figure 7a. The coordination of aluminum in our models was tetrahedral (Al-IV), which is commonly observed in high-pH conditions characteristic of geopolymerization.

The C-(A)-S-H(I) (100) surface (Figure 7b) was chosen to model the adsorption behavior of the oligomers (O_L1_, O_L2_, O_C1_, O_C2_, and O_C3_) (see supplementary materials). Calcium silicate hydrate is widely used as a model of the basic binding phase in concrete. It is a structurally imperfect form of tobermorite, which has a variable composition and length of (alumo) silicate anions [48]. To ensure the isolation of the modeled system and prevent unintended interactions, a buffer zone of 40 Å was incorporated into the model. This approach effectively negates the influence of periodic boundary conditions on the atomic layers within the simulation.

Quantum chemical computations for the gas phase were executed using the DMol3 computational package [49]. The structural optimization of O_L_ and O_C_ oligomers was performed employing an exchange-correlation functional within the framework of the generalized gradient approximation (GGA) [50], specifically utilizing the Perdew–Burke–Ernzerhof (PBE) formulation [51]. PBE offers a good balance between accuracy and computational efficiency. The accuracy of PBE can vary depending on the system being studied. For metallic bulk and surface systems, the PBEsol variant has been shown to provide improved accuracy for some properties. It provides reasonably accurate results for many properties without requiring excessive computational resources, making it a popular choice for various applications [52,53]. Hybrid functionals that incorporate a fraction of Hartree–Fock exchange, such as PBE0, HSE, and B3LYP, can offer improved accuracy for certain properties, particularly for molecular systems [54,55,56]. However, this comes at the cost of increased computational demands. In summary, the PBE functional offers a good compromise between accuracy and computational efficiency for many systems. While more advanced functionals or methods may provide higher accuracy in some cases, they often come with increased computational costs.

The electron density was calculated using a comprehensive set of numerical basis functions with dual polarization capabilities (DNP+) [57]. To enhance the accuracy in describing hydrogen bonding and van der Waals dispersion forces, which are crucial in the hydration process of mineral surfaces [58], a DFT-D correction functional was applied. In this study, the dispersion correction (DFT-D2) was implemented using the method developed by Grimme [59]. The optimization procedure adhered to the following convergence criteria: the energy convergence threshold was set at 1 ×10 ^−6^ Ha, the maximum force tolerance was 2 × 10^−3^ Ha×Å^−1^, and the maximum displacement was limited to 5 × 10^−3^ Å. The self-consistent field (SCF) calculations were performed with a precision of 1 × 10^−6^.

The adsorption process of oligomers on the C-(A)-S-H(I) (100) surface was investigated using stochastic modeling techniques in the gas phase. Optimized structures of oligomers were used for the calculation. During the modeling process, the C-(A)-S-H(I) (100) surface is considered non-deformable. These simulations were carried out using the Adsorption Locator module within the Materials Studio software suite [49]. The computational framework for the model is grounded in the Metropolis algorithm [60]. For the stochastic modeling, a simulated annealing protocol was employed, consisting of 20 temperature cycles with 2 × 10^5^ steps per cycle. The COMPASS force field was utilized to compute energies and identify equilibrium configurations [61,62]. Electrostatic interactions were described using the Ewald summation method, with a precision of 1 × 10^−4^ kcal/mol [63]. The van der Waals interaction energies were evaluated using an atom-based approach. The cutoff radius for Lennard–Jones interactions was set at 14.5 Å. To achieve system equilibrium, an initial 1 × 10^5^ steps were performed, followed by an additional 1 × 10^6^ steps for subsequent calculations.

## 4. Conclusions

In this study, quantum mechanical calculations and stochastic modeling techniques were used to investigate the adsorption behavior and interaction mechanisms of aluminosilicate oligomers with varying Si/Al ratios forming the geopolymer gel structure and calcium silicate hydrate as a substrate at the interface bond region. These oligomers, characterized by varying Si/Al ratios, form the geopolymer gel structure and were studied in relation to calcium silicate hydrate, which served as a substrate at the interface bond region. Furthermore, we determined the energetically favorable structural configurations of oligomers adsorbed onto the C-(A)-S-H(I) (100) surface and computed their respective adsorption energies. The quantum mechanical assessment of local and global reactivity descriptors for aluminosilicate oligomers revealed an increasing adhesion effect correlating with higher Si/Al ratios. This enhancement can be attributed to a proliferation of reactive sites within the atomic structure. Notably, ring-form oligomers demonstrated superior activity compared to their linear counterparts. Our stochastic simulations indicated that the oligomers can be effectively adsorbed onto the calcium silicate hydrate surface through chemisorption processes. We observed that the magnitude of adsorption energies for these oligomers on the studied surface increased proportionally with higher Si/Al ratios, following the sequence: O_C3_ > O_C2_ > O_C1_ > O_L2_ > O_L1_. Ring-shaped oligomers exhibited the highest adsorption capacity with respect to the calcium silicate hydrate surface, suggesting their potential for achieving the most pronounced adhesion effect.

It is worth noting that these computational findings align well with existing experimental studies in the field. This congruence between theoretical predictions and empirical observations lends credence to the validity and practical applicability of our results. 

In conclusion, this study provides valuable molecular-level insights into the adhesion mechanisms at the interface between geopolymer binders and cement mortar. These results contribute to a deeper understanding of the adhesion properties of geopolymers and underscore their potential as effective repair materials in concrete applications.

## Figures and Tables

**Table 1:** Interatomic distances (Å) between the surface layer atoms and the nearest oligomer atoms, as determined by stochastic modeling.

	Al	Si	Si	Si
O_L1_	2.855	1.690		
O_L2_	4.227	1.897	2.036	
O_C1_	2.859	1.677	2.414	4.0241
O_C2_	3.123	1.941	2.353	2.5287
O_C3_	3.234	2.333	1.821	1.7913

## Data Availability

The original contributions presented in the study are included in the article/supplementary material, further inquiries can be directed to the corresponding author.

## Supplementary Materials

The following supporting information can be downloaded at: https://www.mdpi.com/article/10.3390/ijms25158374/s1.
