# Supplementary material for: Molecular Insights into Adhesion at Interface of Geopolymer Binder and Cement Mortar
Source: Int J Mol Sci. 2024 Jul 31;25(15):8374. doi: 10.3390/ijms25158374 (PMC11313433; doi:10.3390/ijms25158374)
Supplement: Supplementary file 1 [file ijms-25-08374-s001.zip › ijms-3075197-supplementary.pdf]

Supplementary Materials

Surface model of the C-(A)-S-H(I)

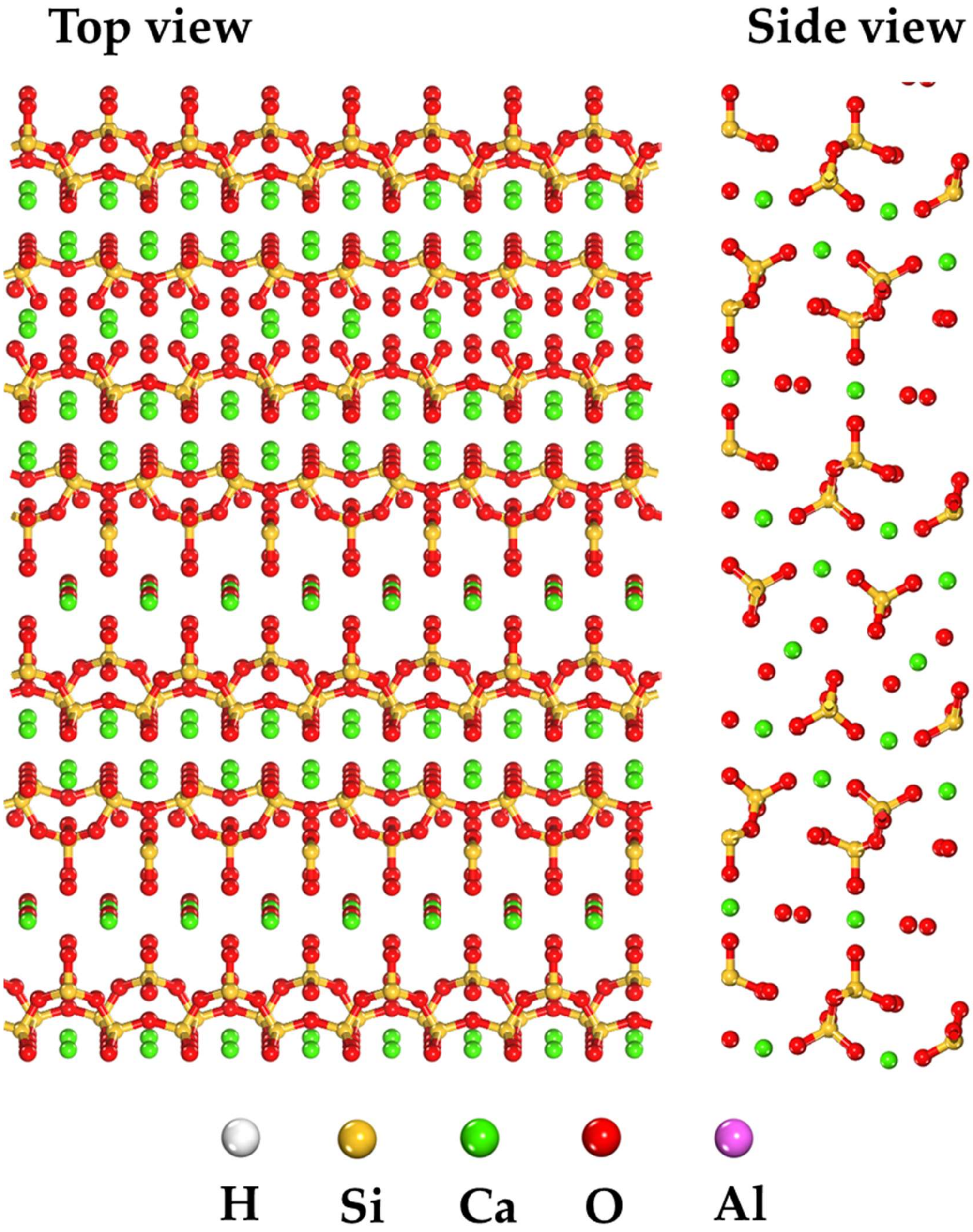

_symmetry_equiv_pos_as_xyz
x,y,z
_cell_length_a 29.2000
_cell_length_b 52.7000
_cell_length_c 60.3566
_cell_angle_alpha 90.0000
_cell_angle_beta 90.0000
_cell_angle_gamma 90.0000
loop_
_atom_site_label
_atom_site_type_symbol

_atom_site_fract_x
_atom_site_fract_y
_atom_site_fract_z
_atom_site_U_iso_or_equiv
_atom_site_adp_type
_atom_site_occupancy
Ca1 Ca 0.03125 0.99397 0.05305 0.00000 Uiso 1.00
Ca2 Ca 0.09375 0.72067 0.05305 0.00000 Uiso 1.00
Ca3 Ca 0.15625 0.99397 0.05305 0.00000 Uiso 1.00
Ca4 Ca 0.21875 0.72067 0.05305 0.00000 Uiso 1.00
Ca5 Ca 0.03125 0.75347 0.10295 0.00000 Uiso 1.00
Ca6 Ca 0.09375 0.57317 0.10295 0.00000 Uiso 1.00
Ca7 Ca 0.15625 0.75347 0.10295 0.00000 Uiso 1.00
Ca8 Ca 0.21875 0.57317 0.10295 0.00000 Uiso 1.00
Ca9 Ca 0.03125 0.54047 0.05305 0.00000 Uiso 1.00
Ca10 Ca 0.09375 0.26717 0.05305 0.00000 Uiso 1.00
Ca11 Ca 0.15625 0.54047 0.05305 0.00000 Uiso 1.00
Ca12 Ca 0.21875 0.26717 0.05305 0.00000 Uiso 1.00
Ca13 Ca 0.03125 0.29997 0.10295 0.00000 Uiso 1.00
Ca14 Ca 0.09375 0.02667 0.10295 0.00000 Uiso 1.00
Ca15 Ca 0.15625 0.29997 0.10295 0.00000 Uiso 1.00
Ca16 Ca 0.21875 0.02667 0.10295 0.00000 Uiso 1.00
Ca17 Ca 0.09375 0.87372 0.07800 0.00000 Uiso 0.25
Ca18 Ca 0.21875 0.87372 0.07800 0.00000 Uiso 0.25
Ca19 Ca 0.03125 0.14692 0.07800 0.00000 Uiso 0.25
Ca20 Ca 0.09375 0.42022 0.07800 0.00000 Uiso 0.25
Ca21 Ca 0.15625 0.14692 0.07800 0.00000 Uiso 0.25
Ca22 Ca 0.21875 0.42022 0.07800 0.00000 Uiso 0.25
Ca23 Ca 0.03125 0.65348 0.03120 0.00000 Uiso 0.25
Ca24 Ca 0.15625 0.65348 0.03120 0.00000 Uiso 0.25
O25 O 0.11323 0.80674 0.06241 0.00000 Uiso 1.00
O26 O 0.20192 0.35324 0.06241 0.00000 Uiso 1.00
O27 O 0.07692 0.48710 0.09359 0.00000 Uiso 1.00
O28 O 0.23822 0.94060 0.09359 0.00000 Uiso 1.00
O29 O 0.01178 0.08004 0.06241 0.00000 Uiso 1.00
O30 O 0.11323 0.35324 0.06241 0.00000 Uiso 1.00
O31 O 0.17308 0.08004 0.06241 0.00000 Uiso 1.00
O32 O 0.20192 0.80674 0.06241 0.00000 Uiso 1.00
O33 O 0.04808 0.21390 0.09359 0.00000 Uiso 1.00
O34 O 0.07692 0.94060 0.09359 0.00000 Uiso 1.00
O35 O 0.13677 0.21390 0.09359 0.00000 Uiso 1.00
O36 O 0.23822 0.48710 0.09359 0.00000 Uiso 1.00
O37 O 0.03125 0.25486 0.12120 0.00000 Uiso 1.00
O38 O 0.09375 0.98166 0.12120 0.00000 Uiso 1.00
O39 O 0.15625 0.70836 0.12120 0.00000 Uiso 1.00
O40 O 0.21875 0.98166 0.12120 0.00000 Uiso 1.00
O41 O 0.03125 0.58549 0.03480 0.00000 Uiso 1.00
O42 O 0.09375 0.76579 0.03480 0.00000 Uiso 1.00
O43 O 0.15625 0.03899 0.03480 0.00000 Uiso 1.00
O44 O 0.21875 0.76579 0.03480 0.00000 Uiso 1.00
O45 O 0.03125 0.70836 0.12120 0.00000 Uiso 1.00
O46 O 0.09375 0.52816 0.12120 0.00000 Uiso 1.00
O47 O 0.15625 0.25486 0.12120 0.00000 Uiso 1.00
O48 O 0.21875 0.52816 0.12120 0.00000 Uiso 1.00
O49 O 0.03125 0.03899 0.03480 0.00000 Uiso 1.00
O50 O 0.09375 0.31229 0.03480 0.00000 Uiso 1.00
O51 O 0.15625 0.58549 0.03480 0.00000 Uiso 1.00
O52 O 0.21875 0.31229 0.03480 0.00000 Uiso 1.00

O53	O	0.03125	0.03422	0.07800	0.00000	Uiso	1.00
O54	O	0.03125	0.25962	0.07800	0.00000	Uiso	1.00
O55	O	0.03125	0.58072	0.07800	0.00000	Uiso	1.00
O56	O	0.03125	0.71312	0.07800	0.00000	Uiso	1.00
O57	O	0.09375	0.30752	0.07800	0.00000	Uiso	1.00
O58	O	0.09375	0.53292	0.07800	0.00000	Uiso	1.00
O59	O	0.09375	0.76102	0.07800	0.00000	Uiso	1.00
O60	O	0.09375	0.98642	0.07800	0.00000	Uiso	1.00
O61	O	0.15625	0.03422	0.07800	0.00000	Uiso	1.00
O62	O	0.15625	0.25962	0.07800	0.00000	Uiso	1.00
O63	O	0.15625	0.58072	0.07800	0.00000	Uiso	1.00
O64	O	0.15625	0.71312	0.07800	0.00000	Uiso	1.00
O65	O	0.21875	0.30752	0.07800	0.00000	Uiso	1.00
O66	O	0.21875	0.53292	0.07800	0.00000	Uiso	1.00
O67	O	0.21875	0.76102	0.07800	0.00000	Uiso	1.00
O68	O	0.21875	0.98642	0.07800	0.00000	Uiso	1.00
O69	O	0.03125	0.33535	0.05776	0.00000	Uiso	1.00
O70	O	0.03125	0.78885	0.05776	0.00000	Uiso	1.00
O71	O	0.09375	0.06205	0.05776	0.00000	Uiso	1.00
O72	O	0.09375	0.60855	0.05776	0.00000	Uiso	1.00
O73	O	0.15625	0.50509	0.09823	0.00000	Uiso	1.00
O74	O	0.15625	0.95859	0.09823	0.00000	Uiso	1.00
O75	O	0.21875	0.23179	0.09823	0.00000	Uiso	1.00
O76	O	0.21875	0.68529	0.09823	0.00000	Uiso	1.00
O77	O	0.01178	0.62654	0.06241	0.00000	Uiso	1.00
O78	O	0.17308	0.62654	0.06241	0.00000	Uiso	1.00
O79	O	0.04808	0.66740	0.09359	0.00000	Uiso	1.00
O80	O	0.13677	0.66740	0.09359	0.00000	Uiso	1.00
O81	O	0.03125	0.44912	0.07800	0.00000	Uiso	1.00
O82	O	0.03125	0.90262	0.07800	0.00000	Uiso	1.00
O83	O	0.09375	0.17592	0.07800	0.00000	Uiso	1.00
O84	O	0.15625	0.39122	0.07800	0.00000	Uiso	1.00
O85	O	0.15625	0.84472	0.07800	0.00000	Uiso	1.00
O86	O	0.21875	0.11802	0.07800	0.00000	Uiso	1.00
O87	O	0.03125	0.81266	0.00874	0.00000	Uiso	1.00
O88	O	0.15625	0.93468	0.14726	0.00000	Uiso	1.00
O89	O	0.03125	0.35916	0.00874	0.00000	Uiso	1.00
O90	O	0.09375	0.08596	0.00874	0.00000	Uiso	1.00
O91	O	0.15625	0.48118	0.14726	0.00000	Uiso	1.00
O92	O	0.21875	0.20798	0.14726	0.00000	Uiso	1.00
O93	O	0.03125	0.39122	0.07800	0.00000	Uiso	0.25
O94	O	0.03125	0.84472	0.07800	0.00000	Uiso	0.25
O95	O	0.09375	0.11802	0.07800	0.00000	Uiso	0.25
O96	O	0.15625	0.44912	0.07800	0.00000	Uiso	0.25
O97	O	0.15625	0.90262	0.07800	0.00000	Uiso	0.25
O98	O	0.21875	0.17592	0.07800	0.00000	Uiso	0.25
O99	O	0.09375	0.63215	0.01155	0.00000	Uiso	1.00
O100	O	0.21875	0.63215	0.01155	0.00000	Uiso	1.00
O101	O	0.09375	0.66179	0.14445	0.00000	Uiso	1.00
O102	O	0.21875	0.66179	0.14445	0.00000	Uiso	1.00
O103	O	0.03125	0.48176	0.14203	0.00000	Uiso	1.00
O104	O	0.03125	0.93526	0.14203	0.00000	Uiso	1.00
O105	O	0.09375	0.20856	0.14203	0.00000	Uiso	1.00
O106	O	0.15625	0.35858	0.01396	0.00000	Uiso	1.00
O107	O	0.15625	0.81208	0.01396	0.00000	Uiso	1.00
O108	O	0.21875	0.08538	0.01396	0.00000	Uiso	1.00
O109	O	0.03125	0.15287	0.02409	0.00000	Uiso	0.25
O110	O	0.09375	0.42617	0.02409	0.00000	Uiso	0.25

O111	O	0.09375	0.87967	0.02409	0.00000	Uiso	0.25
O112	O	0.15625	0.15130	0.03832	0.00000	Uiso	0.25
O113	O	0.21875	0.42460	0.03832	0.00000	Uiso	0.25
O114	O	0.21875	0.87810	0.03832	0.00000	Uiso	0.25
O115	O	0.03125	0.14254	0.11768	0.00000	Uiso	0.25
O116	O	0.09375	0.41584	0.11768	0.00000	Uiso	0.25
O117	O	0.09375	0.86934	0.11768	0.00000	Uiso	0.25
O118	O	0.15625	0.14098	0.13191	0.00000	Uiso	0.25
O119	O	0.21875	0.41428	0.13191	0.00000	Uiso	0.25
O120	O	0.21875	0.86778	0.13191	0.00000	Uiso	0.25
Si121	Si	0.04075	0.05333	0.05795	0.00000	Uiso	1.00
Si122	Si	0.04075	0.59983	0.05795	0.00000	Uiso	1.00
Si123	Si	0.08425	0.32653	0.05795	0.00000	Uiso	1.00
Si124	Si	0.08425	0.78003	0.05795	0.00000	Uiso	1.00
Si125	Si	0.16575	0.24061	0.09805	0.00000	Uiso	1.00
Si126	Si	0.16575	0.69411	0.09805	0.00000	Uiso	1.00
Si127	Si	0.20925	0.51381	0.09805	0.00000	Uiso	1.00
Si128	Si	0.20925	0.96731	0.09805	0.00000	Uiso	1.00
Si129	Si	0.03125	0.47972	0.07800	0.00000	Uiso	1.00
Si130	Si	0.03125	0.93322	0.07800	0.00000	Uiso	1.00
Si131	Si	0.09375	0.20652	0.07800	0.00000	Uiso	1.00
Si132	Si	0.15625	0.36062	0.07800	0.00000	Uiso	1.00
Si133	Si	0.15625	0.81412	0.07800	0.00000	Uiso	1.00
Si134	Si	0.21875	0.08742	0.07800	0.00000	Uiso	1.00
Si135	Si	0.14583	0.05357	0.05849	0.00000	Uiso	1.00
Si136	Si	0.14583	0.60007	0.05849	0.00000	Uiso	1.00
Si137	Si	0.22917	0.32677	0.05849	0.00000	Uiso	1.00
Si138	Si	0.22917	0.78037	0.05849	0.00000	Uiso	1.00
Si139	Si	0.02083	0.24027	0.09750	0.00000	Uiso	1.00
Si140	Si	0.02083	0.69387	0.09750	0.00000	Uiso	1.00
Si141	Si	0.10418	0.51357	0.09750	0.00000	Uiso	1.00
Si142	Si	0.10418	0.96707	0.09750	0.00000	Uiso	1.00
Ca143	Ca	0.15625	0.98365	0.14664	0.00000	Uiso	1.00
Ca144	Ca	0.21875	0.71035	0.14664	0.00000	Uiso	1.00
Ca145	Ca	0.03125	0.98365	0.14664	0.00000	Uiso	1.00
Ca146	Ca	0.09375	0.71035	0.14664	0.00000	Uiso	1.00
Ca147	Ca	0.15625	0.76379	0.00936	0.00000	Uiso	1.00
Ca148	Ca	0.21875	0.58349	0.00936	0.00000	Uiso	1.00
Ca149	Ca	0.03125	0.76379	0.00936	0.00000	Uiso	1.00
Ca150	Ca	0.09375	0.58349	0.00936	0.00000	Uiso	1.00
Ca151	Ca	0.15625	0.53015	0.14664	0.00000	Uiso	1.00
Ca152	Ca	0.21875	0.25685	0.14664	0.00000	Uiso	1.00
Ca153	Ca	0.03125	0.53015	0.14664	0.00000	Uiso	1.00
Ca154	Ca	0.09375	0.25685	0.14664	0.00000	Uiso	1.00
Ca155	Ca	0.15625	0.31029	0.00936	0.00000	Uiso	1.00
Ca156	Ca	0.21875	0.03699	0.00936	0.00000	Uiso	1.00
Ca157	Ca	0.03125	0.31029	0.00936	0.00000	Uiso	1.00
Ca158	Ca	0.09375	0.03699	0.00936	0.00000	Uiso	1.00
Ca159	Ca	0.21875	0.86340	0.17159	0.00000	Uiso	0.25
Ca160	Ca	0.09375	0.86340	0.17159	0.00000	Uiso	0.25
Ca161	Ca	0.15625	0.13660	0.17159	0.00000	Uiso	0.25
Ca162	Ca	0.21875	0.40990	0.17159	0.00000	Uiso	0.25
Ca163	Ca	0.03125	0.13660	0.17159	0.00000	Uiso	0.25
Ca164	Ca	0.09375	0.40990	0.17159	0.00000	Uiso	0.25
Ca165	Ca	0.15625	0.64316	0.12480	0.00000	Uiso	0.25
Ca166	Ca	0.03125	0.64316	0.12480	0.00000	Uiso	0.25
O167	O	0.23822	0.79642	0.15600	0.00000	Uiso	1.00
O168	O	0.07692	0.34292	0.15600	0.00000	Uiso	1.00

O169	O	0.20192	0.49742	0.00000	0.00000	Uiso	1.00
O170	O	0.11323	0.95092	0.00000	0.00000	Uiso	1.00
O171	O	0.13677	0.06972	0.15600	0.00000	Uiso	1.00
O172	O	0.23822	0.34292	0.15600	0.00000	Uiso	1.00
O173	O	0.04808	0.06972	0.15600	0.00000	Uiso	1.00
O174	O	0.07692	0.79642	0.15600	0.00000	Uiso	1.00
O175	O	0.17308	0.22422	0.00000	0.00000	Uiso	1.00
O176	O	0.20192	0.95092	0.00000	0.00000	Uiso	1.00
O177	O	0.01178	0.22422	0.00000	0.00000	Uiso	1.00
O178	O	0.11323	0.49742	0.00000	0.00000	Uiso	1.00
O179	O	0.15625	0.26518	0.02761	0.00000	Uiso	1.00
O180	O	0.21875	0.99198	0.02761	0.00000	Uiso	1.00
O181	O	0.03125	0.71868	0.02761	0.00000	Uiso	1.00
O182	O	0.09375	0.99198	0.02761	0.00000	Uiso	1.00
O183	O	0.15625	0.57516	0.12839	0.00000	Uiso	1.00
O184	O	0.21875	0.75546	0.12839	0.00000	Uiso	1.00
O185	O	0.03125	0.02866	0.12839	0.00000	Uiso	1.00
O186	O	0.09375	0.75546	0.12839	0.00000	Uiso	1.00
O187	O	0.15625	0.71868	0.02761	0.00000	Uiso	1.00
O188	O	0.21875	0.53848	0.02761	0.00000	Uiso	1.00
O189	O	0.03125	0.26518	0.02761	0.00000	Uiso	1.00
O190	O	0.09375	0.53848	0.02761	0.00000	Uiso	1.00
O191	O	0.15625	0.02866	0.12839	0.00000	Uiso	1.00
O192	O	0.21875	0.30196	0.12839	0.00000	Uiso	1.00
O193	O	0.03125	0.57516	0.12839	0.00000	Uiso	1.00
O194	O	0.09375	0.30196	0.12839	0.00000	Uiso	1.00
O195	O	0.15625	0.02390	0.17159	0.00000	Uiso	1.00
O196	O	0.15625	0.24930	0.17159	0.00000	Uiso	1.00
O197	O	0.15625	0.57040	0.17159	0.00000	Uiso	1.00
O198	O	0.15625	0.70280	0.17159	0.00000	Uiso	1.00
O199	O	0.21875	0.29720	0.17159	0.00000	Uiso	1.00
O200	O	0.21875	0.52260	0.17159	0.00000	Uiso	1.00
O201	O	0.21875	0.75070	0.17159	0.00000	Uiso	1.00
O202	O	0.21875	0.97610	0.17159	0.00000	Uiso	1.00
O203	O	0.03125	0.02390	0.17159	0.00000	Uiso	1.00
O204	O	0.03125	0.24930	0.17159	0.00000	Uiso	1.00
O205	O	0.03125	0.57040	0.17159	0.00000	Uiso	1.00
O206	O	0.03125	0.70280	0.17159	0.00000	Uiso	1.00
O207	O	0.09375	0.29720	0.17159	0.00000	Uiso	1.00
O208	O	0.09375	0.52260	0.17159	0.00000	Uiso	1.00
O209	O	0.09375	0.75070	0.17159	0.00000	Uiso	1.00
O210	O	0.09375	0.97610	0.17159	0.00000	Uiso	1.00
O211	O	0.15625	0.32503	0.15136	0.00000	Uiso	1.00
O212	O	0.15625	0.77853	0.15136	0.00000	Uiso	1.00
O213	O	0.21875	0.05173	0.15136	0.00000	Uiso	1.00
O214	O	0.21875	0.59823	0.15136	0.00000	Uiso	1.00
O215	O	0.03125	0.51541	0.00464	0.00000	Uiso	1.00
O216	O	0.03125	0.96891	0.00464	0.00000	Uiso	1.00
O217	O	0.09375	0.24211	0.00464	0.00000	Uiso	1.00
O218	O	0.09375	0.69561	0.00464	0.00000	Uiso	1.00
O219	O	0.13677	0.61622	0.15600	0.00000	Uiso	1.00
O220	O	0.04808	0.61622	0.15600	0.00000	Uiso	1.00
O221	O	0.17308	0.67772	0.00000	0.00000	Uiso	1.00
O222	O	0.01178	0.67772	0.00000	0.00000	Uiso	1.00
O223	O	0.15625	0.43880	0.17159	0.00000	Uiso	1.00
O224	O	0.15625	0.89230	0.17159	0.00000	Uiso	1.00
O225	O	0.21875	0.16560	0.17159	0.00000	Uiso	1.00
O226	O	0.03125	0.38090	0.17159	0.00000	Uiso	1.00

O227	O	0.03125	0.83440	0.17159	0.00000	Uiso	1.00
O228	O	0.09375	0.10770	0.17159	0.00000	Uiso	1.00
O229	O	0.15625	0.80234	0.10233	0.00000	Uiso	1.00
O230	O	0.03125	0.94500	0.05367	0.00000	Uiso	1.00
O231	O	0.15625	0.34884	0.10233	0.00000	Uiso	1.00
O232	O	0.21875	0.07564	0.10233	0.00000	Uiso	1.00
O233	O	0.03125	0.49150	0.05367	0.00000	Uiso	1.00
O234	O	0.09375	0.21830	0.05367	0.00000	Uiso	1.00
O235	O	0.15625	0.38090	0.17159	0.00000	Uiso	0.25
O236	O	0.15625	0.83440	0.17159	0.00000	Uiso	0.25
O237	O	0.21875	0.10770	0.17159	0.00000	Uiso	0.25
O238	O	0.03125	0.43880	0.17159	0.00000	Uiso	0.25
O239	O	0.03125	0.89230	0.17159	0.00000	Uiso	0.25
O240	O	0.09375	0.16560	0.17159	0.00000	Uiso	0.25
O241	O	0.21875	0.62183	0.10514	0.00000	Uiso	1.00
O242	O	0.09375	0.62183	0.10514	0.00000	Uiso	1.00
O243	O	0.21875	0.67211	0.05086	0.00000	Uiso	1.00
O244	O	0.09375	0.67211	0.05086	0.00000	Uiso	1.00
O245	O	0.15625	0.49208	0.04844	0.00000	Uiso	1.00
O246	O	0.15625	0.94558	0.04844	0.00000	Uiso	1.00
O247	O	0.21875	0.21888	0.04844	0.00000	Uiso	1.00
O248	O	0.03125	0.34826	0.10756	0.00000	Uiso	1.00
O249	O	0.03125	0.80176	0.10756	0.00000	Uiso	1.00
O250	O	0.09375	0.07506	0.10756	0.00000	Uiso	1.00
O251	O	0.15625	0.14254	0.11768	0.00000	Uiso	0.25
O252	O	0.21875	0.41584	0.11768	0.00000	Uiso	0.25
O253	O	0.21875	0.86934	0.11768	0.00000	Uiso	0.25
O254	O	0.03125	0.14098	0.13191	0.00000	Uiso	0.25
O255	O	0.09375	0.41428	0.13191	0.00000	Uiso	0.25
O256	O	0.09375	0.86778	0.13191	0.00000	Uiso	0.25
O257	O	0.15625	0.15287	0.02409	0.00000	Uiso	0.25
O258	O	0.21875	0.42617	0.02409	0.00000	Uiso	0.25
O259	O	0.21875	0.87967	0.02409	0.00000	Uiso	0.25
O260	O	0.03125	0.15130	0.03832	0.00000	Uiso	0.25
O261	O	0.09375	0.42460	0.03832	0.00000	Uiso	0.25
O262	O	0.09375	0.87810	0.03832	0.00000	Uiso	0.25
Si263	Si	0.16575	0.04301	0.15154	0.00000	Uiso	1.00
Si264	Si	0.16575	0.58951	0.15154	0.00000	Uiso	1.00
Si265	Si	0.20925	0.31621	0.15154	0.00000	Uiso	1.00
Si266	Si	0.20925	0.76971	0.15154	0.00000	Uiso	1.00
Si267	Si	0.04075	0.25093	0.00445	0.00000	Uiso	1.00
Si268	Si	0.04075	0.70443	0.00445	0.00000	Uiso	1.00
Si269	Si	0.08425	0.52413	0.00445	0.00000	Uiso	1.00
Si270	Si	0.08425	0.97763	0.00445	0.00000	Uiso	1.00
Si271	Si	0.15625	0.46940	0.17159	0.00000	Uiso	1.00
Si272	Si	0.15625	0.92290	0.17159	0.00000	Uiso	1.00
Si273	Si	0.21875	0.19620	0.17159	0.00000	Uiso	1.00
Si274	Si	0.03125	0.35030	0.17159	0.00000	Uiso	1.00
Si275	Si	0.03125	0.80380	0.17159	0.00000	Uiso	1.00
Si276	Si	0.09375	0.07710	0.17159	0.00000	Uiso	1.00
Si277	Si	0.02082	0.04325	0.15209	0.00000	Uiso	1.00
Si278	Si	0.02082	0.58975	0.15209	0.00000	Uiso	1.00
Si279	Si	0.10418	0.31645	0.15209	0.00000	Uiso	1.00
Si280	Si	0.10418	0.77005	0.15209	0.00000	Uiso	1.00
Si281	Si	0.14583	0.25059	0.00391	0.00000	Uiso	1.00
Si282	Si	0.14583	0.70419	0.00391	0.00000	Uiso	1.00
Si283	Si	0.22917	0.52389	0.00391	0.00000	Uiso	1.00
Si284	Si	0.22917	0.97739	0.00391	0.00000	Uiso	1.00

Ca285	Ca	0.28125	0.99397	0.05305	0.00000	Uiso	1.00
Ca286	Ca	0.34375	0.72067	0.05305	0.00000	Uiso	1.00
Ca287	Ca	0.40625	0.99397	0.05305	0.00000	Uiso	1.00
Ca288	Ca	0.46875	0.72067	0.05305	0.00000	Uiso	1.00
Ca289	Ca	0.28125	0.75347	0.10295	0.00000	Uiso	1.00
Ca290	Ca	0.34375	0.57317	0.10295	0.00000	Uiso	1.00
Ca291	Ca	0.40625	0.75347	0.10295	0.00000	Uiso	1.00
Ca292	Ca	0.46875	0.57317	0.10295	0.00000	Uiso	1.00
Ca293	Ca	0.28125	0.54047	0.05305	0.00000	Uiso	1.00
Ca294	Ca	0.34375	0.26717	0.05305	0.00000	Uiso	1.00
Ca295	Ca	0.40625	0.54047	0.05305	0.00000	Uiso	1.00
Ca296	Ca	0.46875	0.26717	0.05305	0.00000	Uiso	1.00
Ca297	Ca	0.28125	0.29997	0.10295	0.00000	Uiso	1.00
Ca298	Ca	0.34375	0.02667	0.10295	0.00000	Uiso	1.00
Ca299	Ca	0.40625	0.29997	0.10295	0.00000	Uiso	1.00
Ca300	Ca	0.46875	0.02667	0.10295	0.00000	Uiso	1.00
Ca301	Ca	0.34375	0.87372	0.07800	0.00000	Uiso	0.25
Ca302	Ca	0.46875	0.87372	0.07800	0.00000	Uiso	0.25
Ca303	Ca	0.28125	0.14692	0.07800	0.00000	Uiso	0.25
Ca304	Ca	0.34375	0.42022	0.07800	0.00000	Uiso	0.25
Ca305	Ca	0.40625	0.14692	0.07800	0.00000	Uiso	0.25
Ca306	Ca	0.46875	0.42022	0.07800	0.00000	Uiso	0.25
Ca307	Ca	0.28125	0.65348	0.03120	0.00000	Uiso	0.25
Ca308	Ca	0.40625	0.65348	0.03120	0.00000	Uiso	0.25
O309	O	0.36323	0.80674	0.06241	0.00000	Uiso	1.00
O310	O	0.45193	0.35324	0.06241	0.00000	Uiso	1.00
O311	O	0.32692	0.48710	0.09359	0.00000	Uiso	1.00
O312	O	0.48823	0.94060	0.09359	0.00000	Uiso	1.00
O313	O	0.26177	0.08004	0.06241	0.00000	Uiso	1.00
O314	O	0.36323	0.35324	0.06241	0.00000	Uiso	1.00
O315	O	0.42307	0.08004	0.06241	0.00000	Uiso	1.00
O316	O	0.45193	0.80674	0.06241	0.00000	Uiso	1.00
O317	O	0.29807	0.21390	0.09359	0.00000	Uiso	1.00
O318	O	0.32692	0.94060	0.09359	0.00000	Uiso	1.00
O319	O	0.38677	0.21390	0.09359	0.00000	Uiso	1.00
O320	O	0.48823	0.48710	0.09359	0.00000	Uiso	1.00
O321	O	0.28125	0.25486	0.12120	0.00000	Uiso	1.00
O322	O	0.34375	0.98166	0.12120	0.00000	Uiso	1.00
O323	O	0.40625	0.70836	0.12120	0.00000	Uiso	1.00
O324	O	0.46875	0.98166	0.12120	0.00000	Uiso	1.00
O325	O	0.28125	0.58549	0.03480	0.00000	Uiso	1.00
O326	O	0.34375	0.76579	0.03480	0.00000	Uiso	1.00
O327	O	0.40625	0.03899	0.03480	0.00000	Uiso	1.00
O328	O	0.46875	0.76579	0.03480	0.00000	Uiso	1.00
O329	O	0.28125	0.70836	0.12120	0.00000	Uiso	1.00
O330	O	0.34375	0.52816	0.12120	0.00000	Uiso	1.00
O331	O	0.40625	0.25486	0.12120	0.00000	Uiso	1.00
O332	O	0.46875	0.52816	0.12120	0.00000	Uiso	1.00
O333	O	0.28125	0.03899	0.03480	0.00000	Uiso	1.00
O334	O	0.34375	0.31229	0.03480	0.00000	Uiso	1.00
O335	O	0.40625	0.58549	0.03480	0.00000	Uiso	1.00
O336	O	0.46875	0.31229	0.03480	0.00000	Uiso	1.00
O337	O	0.28125	0.03422	0.07800	0.00000	Uiso	1.00
O338	O	0.28125	0.25962	0.07800	0.00000	Uiso	1.00
O339	O	0.28125	0.58072	0.07800	0.00000	Uiso	1.00
O340	O	0.28125	0.71312	0.07800	0.00000	Uiso	1.00
O341	O	0.34375	0.30752	0.07800	0.00000	Uiso	1.00
O342	O	0.34375	0.53292	0.07800	0.00000	Uiso	1.00

O343	O	0.34375	0.76102	0.07800	0.00000	Uiso	1.00
O344	O	0.34375	0.98642	0.07800	0.00000	Uiso	1.00
O345	O	0.40625	0.03422	0.07800	0.00000	Uiso	1.00
O346	O	0.40625	0.25962	0.07800	0.00000	Uiso	1.00
O347	O	0.40625	0.58072	0.07800	0.00000	Uiso	1.00
O348	O	0.40625	0.71312	0.07800	0.00000	Uiso	1.00
O349	O	0.46875	0.30752	0.07800	0.00000	Uiso	1.00
O350	O	0.46875	0.53292	0.07800	0.00000	Uiso	1.00
O351	O	0.46875	0.76102	0.07800	0.00000	Uiso	1.00
O352	O	0.46875	0.98642	0.07800	0.00000	Uiso	1.00
O353	O	0.28125	0.33535	0.05776	0.00000	Uiso	1.00
O354	O	0.28125	0.78885	0.05776	0.00000	Uiso	1.00
O355	O	0.34375	0.06205	0.05776	0.00000	Uiso	1.00
O356	O	0.34375	0.60855	0.05776	0.00000	Uiso	1.00
O357	O	0.40625	0.50509	0.09823	0.00000	Uiso	1.00
O358	O	0.40625	0.95859	0.09823	0.00000	Uiso	1.00
O359	O	0.46875	0.23179	0.09823	0.00000	Uiso	1.00
O360	O	0.46875	0.68529	0.09823	0.00000	Uiso	1.00
O361	O	0.26177	0.62654	0.06241	0.00000	Uiso	1.00
O362	O	0.42307	0.62654	0.06241	0.00000	Uiso	1.00
O363	O	0.29807	0.66740	0.09359	0.00000	Uiso	1.00
O364	O	0.38677	0.66740	0.09359	0.00000	Uiso	1.00
O365	O	0.28125	0.44912	0.07800	0.00000	Uiso	1.00
O366	O	0.28125	0.90262	0.07800	0.00000	Uiso	1.00
O367	O	0.34375	0.17592	0.07800	0.00000	Uiso	1.00
O368	O	0.40625	0.39122	0.07800	0.00000	Uiso	1.00
O369	O	0.40625	0.84472	0.07800	0.00000	Uiso	1.00
O370	O	0.46875	0.11802	0.07800	0.00000	Uiso	1.00
O371	O	0.28125	0.81266	0.00874	0.00000	Uiso	1.00
O372	O	0.40625	0.93468	0.14726	0.00000	Uiso	1.00
O373	O	0.28125	0.35916	0.00874	0.00000	Uiso	1.00
O374	O	0.34375	0.08596	0.00874	0.00000	Uiso	1.00
O375	O	0.40625	0.48118	0.14726	0.00000	Uiso	1.00
O376	O	0.46875	0.20798	0.14726	0.00000	Uiso	1.00
O377	O	0.28125	0.39122	0.07800	0.00000	Uiso	0.25
O378	O	0.28125	0.84472	0.07800	0.00000	Uiso	0.25
O379	O	0.34375	0.11802	0.07800	0.00000	Uiso	0.25
O380	O	0.40625	0.44912	0.07800	0.00000	Uiso	0.25
O381	O	0.40625	0.90262	0.07800	0.00000	Uiso	0.25
O382	O	0.46875	0.17592	0.07800	0.00000	Uiso	0.25
O383	O	0.34375	0.63215	0.01155	0.00000	Uiso	1.00
O384	O	0.46875	0.63215	0.01155	0.00000	Uiso	1.00
O385	O	0.34375	0.66179	0.14445	0.00000	Uiso	1.00
O386	O	0.46875	0.66179	0.14445	0.00000	Uiso	1.00
O387	O	0.28125	0.48176	0.14203	0.00000	Uiso	1.00
O388	O	0.28125	0.93526	0.14203	0.00000	Uiso	1.00
O389	O	0.34375	0.20856	0.14203	0.00000	Uiso	1.00
O390	O	0.40625	0.35858	0.01396	0.00000	Uiso	1.00
O391	O	0.40625	0.81208	0.01396	0.00000	Uiso	1.00
O392	O	0.46875	0.08538	0.01396	0.00000	Uiso	1.00
O393	O	0.28125	0.15287	0.02409	0.00000	Uiso	0.25
O394	O	0.34375	0.42617	0.02409	0.00000	Uiso	0.25
O395	O	0.34375	0.87967	0.02409	0.00000	Uiso	0.25
O396	O	0.40625	0.15130	0.03832	0.00000	Uiso	0.25
O397	O	0.46875	0.42460	0.03832	0.00000	Uiso	0.25
O398	O	0.46875	0.87810	0.03832	0.00000	Uiso	0.25
O399	O	0.28125	0.14254	0.11768	0.00000	Uiso	0.25
O400	O	0.34375	0.41584	0.11768	0.00000	Uiso	0.25

O401	O	0.34375	0.86934	0.11768	0.00000	Uiso	0.25
O402	O	0.40625	0.14098	0.13191	0.00000	Uiso	0.25
O403	O	0.46875	0.41428	0.13191	0.00000	Uiso	0.25
O404	O	0.46875	0.86778	0.13191	0.00000	Uiso	0.25
Si405	Si	0.29075	0.05333	0.05795	0.00000	Uiso	1.00
Si406	Si	0.29075	0.59983	0.05795	0.00000	Uiso	1.00
Si407	Si	0.33425	0.32653	0.05795	0.00000	Uiso	1.00
Si408	Si	0.33425	0.78003	0.05795	0.00000	Uiso	1.00
Si409	Si	0.41575	0.24061	0.09805	0.00000	Uiso	1.00
Si410	Si	0.41575	0.69411	0.09805	0.00000	Uiso	1.00
Si411	Si	0.45925	0.51381	0.09805	0.00000	Uiso	1.00
Si412	Si	0.45925	0.96731	0.09805	0.00000	Uiso	1.00
Si413	Si	0.28125	0.47972	0.07800	0.00000	Uiso	1.00
Si414	Si	0.28125	0.93322	0.07800	0.00000	Uiso	1.00
Si415	Si	0.34375	0.20652	0.07800	0.00000	Uiso	1.00
Si416	Si	0.40625	0.36062	0.07800	0.00000	Uiso	1.00
Si417	Si	0.40625	0.81412	0.07800	0.00000	Uiso	1.00
Si418	Si	0.46875	0.08742	0.07800	0.00000	Uiso	1.00
Si419	Si	0.39582	0.05357	0.05849	0.00000	Uiso	1.00
Si420	Si	0.39582	0.60007	0.05849	0.00000	Uiso	1.00
Si421	Si	0.47918	0.32677	0.05849	0.00000	Uiso	1.00
Si422	Si	0.47918	0.78037	0.05849	0.00000	Uiso	1.00
Si423	Si	0.27082	0.24027	0.09750	0.00000	Uiso	1.00
Si424	Si	0.27082	0.69387	0.09750	0.00000	Uiso	1.00
Si425	Si	0.35418	0.51357	0.09750	0.00000	Uiso	1.00
Si426	Si	0.35418	0.96707	0.09750	0.00000	Uiso	1.00
Ca427	Ca	0.40625	0.98365	0.14664	0.00000	Uiso	1.00
Ca428	Ca	0.46875	0.71035	0.14664	0.00000	Uiso	1.00
Ca429	Ca	0.28125	0.98365	0.14664	0.00000	Uiso	1.00
Ca430	Ca	0.34375	0.71035	0.14664	0.00000	Uiso	1.00
Ca431	Ca	0.40625	0.76379	0.00936	0.00000	Uiso	1.00
Ca432	Ca	0.46875	0.58349	0.00936	0.00000	Uiso	1.00
Ca433	Ca	0.28125	0.76379	0.00936	0.00000	Uiso	1.00
Ca434	Ca	0.34375	0.58349	0.00936	0.00000	Uiso	1.00
Ca435	Ca	0.40625	0.53015	0.14664	0.00000	Uiso	1.00
Ca436	Ca	0.46875	0.25685	0.14664	0.00000	Uiso	1.00
Ca437	Ca	0.28125	0.53015	0.14664	0.00000	Uiso	1.00
Ca438	Ca	0.34375	0.25685	0.14664	0.00000	Uiso	1.00
Ca439	Ca	0.40625	0.31029	0.00936	0.00000	Uiso	1.00
Ca440	Ca	0.46875	0.03699	0.00936	0.00000	Uiso	1.00
Ca441	Ca	0.28125	0.31029	0.00936	0.00000	Uiso	1.00
Ca442	Ca	0.34375	0.03699	0.00936	0.00000	Uiso	1.00
Ca443	Ca	0.46875	0.86340	0.17159	0.00000	Uiso	0.25
Ca444	Ca	0.34375	0.86340	0.17159	0.00000	Uiso	0.25
Ca445	Ca	0.40625	0.13660	0.17159	0.00000	Uiso	0.25
Ca446	Ca	0.46875	0.40990	0.17159	0.00000	Uiso	0.25
Ca447	Ca	0.28125	0.13660	0.17159	0.00000	Uiso	0.25
Ca448	Ca	0.34375	0.40990	0.17159	0.00000	Uiso	0.25
Ca449	Ca	0.40625	0.64316	0.12480	0.00000	Uiso	0.25
Ca450	Ca	0.28125	0.64316	0.12480	0.00000	Uiso	0.25
O451	O	0.48823	0.79642	0.15600	0.00000	Uiso	1.00
O452	O	0.32692	0.34292	0.15600	0.00000	Uiso	1.00
O453	O	0.45193	0.49742	0.00000	0.00000	Uiso	1.00
O454	O	0.36323	0.95092	0.00000	0.00000	Uiso	1.00
O455	O	0.38677	0.06972	0.15600	0.00000	Uiso	1.00
O456	O	0.48823	0.34292	0.15600	0.00000	Uiso	1.00
O457	O	0.29807	0.06972	0.15600	0.00000	Uiso	1.00
O458	O	0.32692	0.79642	0.15600	0.00000	Uiso	1.00

O459	O	0.42307	0.22422	0.00000	0.00000	Uiso	1.00
O460	O	0.45193	0.95092	0.00000	0.00000	Uiso	1.00
O461	O	0.26177	0.22422	0.00000	0.00000	Uiso	1.00
O462	O	0.36323	0.49742	0.00000	0.00000	Uiso	1.00
O463	O	0.40625	0.26518	0.02761	0.00000	Uiso	1.00
O464	O	0.46875	0.99198	0.02761	0.00000	Uiso	1.00
O465	O	0.28125	0.71868	0.02761	0.00000	Uiso	1.00
O466	O	0.34375	0.99198	0.02761	0.00000	Uiso	1.00
O467	O	0.40625	0.57516	0.12839	0.00000	Uiso	1.00
O468	O	0.46875	0.75546	0.12839	0.00000	Uiso	1.00
O469	O	0.28125	0.02866	0.12839	0.00000	Uiso	1.00
O470	O	0.34375	0.75546	0.12839	0.00000	Uiso	1.00
O471	O	0.40625	0.71868	0.02761	0.00000	Uiso	1.00
O472	O	0.46875	0.53848	0.02761	0.00000	Uiso	1.00
O473	O	0.28125	0.26518	0.02761	0.00000	Uiso	1.00
O474	O	0.34375	0.53848	0.02761	0.00000	Uiso	1.00
O475	O	0.40625	0.02866	0.12839	0.00000	Uiso	1.00
O476	O	0.46875	0.30196	0.12839	0.00000	Uiso	1.00
O477	O	0.28125	0.57516	0.12839	0.00000	Uiso	1.00
O478	O	0.34375	0.30196	0.12839	0.00000	Uiso	1.00
O479	O	0.40625	0.02390	0.17159	0.00000	Uiso	1.00
O480	O	0.40625	0.24930	0.17159	0.00000	Uiso	1.00
O481	O	0.40625	0.57040	0.17159	0.00000	Uiso	1.00
O482	O	0.40625	0.70280	0.17159	0.00000	Uiso	1.00
O483	O	0.46875	0.29720	0.17159	0.00000	Uiso	1.00
O484	O	0.46875	0.52260	0.17159	0.00000	Uiso	1.00
O485	O	0.46875	0.75070	0.17159	0.00000	Uiso	1.00
O486	O	0.46875	0.97610	0.17159	0.00000	Uiso	1.00
O487	O	0.28125	0.02390	0.17159	0.00000	Uiso	1.00
O488	O	0.28125	0.24930	0.17159	0.00000	Uiso	1.00
O489	O	0.28125	0.57040	0.17159	0.00000	Uiso	1.00
O490	O	0.28125	0.70280	0.17159	0.00000	Uiso	1.00
O491	O	0.34375	0.29720	0.17159	0.00000	Uiso	1.00
O492	O	0.34375	0.52260	0.17159	0.00000	Uiso	1.00
O493	O	0.34375	0.75070	0.17159	0.00000	Uiso	1.00
O494	O	0.34375	0.97610	0.17159	0.00000	Uiso	1.00
O495	O	0.40625	0.32503	0.15136	0.00000	Uiso	1.00
O496	O	0.40625	0.77853	0.15136	0.00000	Uiso	1.00
O497	O	0.46875	0.05173	0.15136	0.00000	Uiso	1.00
O498	O	0.46875	0.59823	0.15136	0.00000	Uiso	1.00
O499	O	0.28125	0.51541	0.00464	0.00000	Uiso	1.00
O500	O	0.28125	0.96891	0.00464	0.00000	Uiso	1.00
O501	O	0.34375	0.24211	0.00464	0.00000	Uiso	1.00
O502	O	0.34375	0.69561	0.00464	0.00000	Uiso	1.00
O503	O	0.38677	0.61622	0.15600	0.00000	Uiso	1.00
O504	O	0.29807	0.61622	0.15600	0.00000	Uiso	1.00
O505	O	0.42307	0.67772	0.00000	0.00000	Uiso	1.00
O506	O	0.26177	0.67772	0.00000	0.00000	Uiso	1.00
O507	O	0.40625	0.43880	0.17159	0.00000	Uiso	1.00
O508	O	0.40625	0.89230	0.17159	0.00000	Uiso	1.00
O509	O	0.46875	0.16560	0.17159	0.00000	Uiso	1.00
O510	O	0.28125	0.38090	0.17159	0.00000	Uiso	1.00
O511	O	0.28125	0.83440	0.17159	0.00000	Uiso	1.00
O512	O	0.34375	0.10770	0.17159	0.00000	Uiso	1.00
O513	O	0.40625	0.80234	0.10233	0.00000	Uiso	1.00
O514	O	0.28125	0.94500	0.05367	0.00000	Uiso	1.00
O515	O	0.40625	0.34884	0.10233	0.00000	Uiso	1.00
O516	O	0.46875	0.07564	0.10233	0.00000	Uiso	1.00

O517	O	0.28125	0.49150	0.05367	0.00000	Uiso	1.00
O518	O	0.34375	0.21830	0.05367	0.00000	Uiso	1.00
O519	O	0.40625	0.38090	0.17159	0.00000	Uiso	0.25
O520	O	0.40625	0.83440	0.17159	0.00000	Uiso	0.25
O521	O	0.46875	0.10770	0.17159	0.00000	Uiso	0.25
O522	O	0.28125	0.43880	0.17159	0.00000	Uiso	0.25
O523	O	0.28125	0.89230	0.17159	0.00000	Uiso	0.25
O524	O	0.34375	0.16560	0.17159	0.00000	Uiso	0.25
O525	O	0.46875	0.62183	0.10514	0.00000	Uiso	1.00
O526	O	0.34375	0.62183	0.10514	0.00000	Uiso	1.00
O527	O	0.46875	0.67211	0.05086	0.00000	Uiso	1.00
O528	O	0.34375	0.67211	0.05086	0.00000	Uiso	1.00
O529	O	0.40625	0.49208	0.04844	0.00000	Uiso	1.00
O530	O	0.40625	0.94558	0.04844	0.00000	Uiso	1.00
O531	O	0.46875	0.21888	0.04844	0.00000	Uiso	1.00
O532	O	0.28125	0.34826	0.10756	0.00000	Uiso	1.00
O533	O	0.28125	0.80176	0.10756	0.00000	Uiso	1.00
O534	O	0.34375	0.07506	0.10756	0.00000	Uiso	1.00
O535	O	0.40625	0.14254	0.11768	0.00000	Uiso	0.25
O536	O	0.46875	0.41584	0.11768	0.00000	Uiso	0.25
O537	O	0.46875	0.86934	0.11768	0.00000	Uiso	0.25
O538	O	0.28125	0.14098	0.13191	0.00000	Uiso	0.25
O539	O	0.34375	0.41428	0.13191	0.00000	Uiso	0.25
O540	O	0.34375	0.86778	0.13191	0.00000	Uiso	0.25
O541	O	0.40625	0.15287	0.02409	0.00000	Uiso	0.25
O542	O	0.46875	0.42617	0.02409	0.00000	Uiso	0.25
O543	O	0.46875	0.87967	0.02409	0.00000	Uiso	0.25
O544	O	0.28125	0.15130	0.03832	0.00000	Uiso	0.25
O545	O	0.34375	0.42460	0.03832	0.00000	Uiso	0.25
O546	O	0.34375	0.87810	0.03832	0.00000	Uiso	0.25
Si547	Si	0.41575	0.04301	0.15154	0.00000	Uiso	1.00
Si548	Si	0.41575	0.58951	0.15154	0.00000	Uiso	1.00
Si549	Si	0.45925	0.31621	0.15154	0.00000	Uiso	1.00
Si550	Si	0.45925	0.76971	0.15154	0.00000	Uiso	1.00
Si551	Si	0.29075	0.25093	0.00445	0.00000	Uiso	1.00
Si552	Si	0.29075	0.70443	0.00445	0.00000	Uiso	1.00
Si553	Si	0.33425	0.52413	0.00445	0.00000	Uiso	1.00
Si554	Si	0.33425	0.97763	0.00445	0.00000	Uiso	1.00
Si555	Si	0.40625	0.46940	0.17159	0.00000	Uiso	1.00
Si556	Si	0.40625	0.92290	0.17159	0.00000	Uiso	1.00
Si557	Si	0.46875	0.19620	0.17159	0.00000	Uiso	1.00
Si558	Si	0.28125	0.35030	0.17159	0.00000	Uiso	1.00
Si559	Si	0.28125	0.80380	0.17159	0.00000	Uiso	1.00
Si560	Si	0.34375	0.07710	0.17159	0.00000	Uiso	1.00
Si561	Si	0.27082	0.04325	0.15209	0.00000	Uiso	1.00
Si562	Si	0.27082	0.58975	0.15209	0.00000	Uiso	1.00
Si563	Si	0.35418	0.31645	0.15209	0.00000	Uiso	1.00
Si564	Si	0.35418	0.77005	0.15209	0.00000	Uiso	1.00
Si565	Si	0.39582	0.25059	0.00391	0.00000	Uiso	1.00
Si566	Si	0.39582	0.70419	0.00391	0.00000	Uiso	1.00
Si567	Si	0.47918	0.52389	0.00391	0.00000	Uiso	1.00
Si568	Si	0.47918	0.97739	0.00391	0.00000	Uiso	1.00
Ca569	Ca	0.53125	0.99397	0.05305	0.00000	Uiso	1.00
Ca570	Ca	0.59375	0.72067	0.05305	0.00000	Uiso	1.00
Ca571	Ca	0.65625	0.99397	0.05305	0.00000	Uiso	1.00
Ca572	Ca	0.71875	0.72067	0.05305	0.00000	Uiso	1.00
Ca573	Ca	0.53125	0.75347	0.10295	0.00000	Uiso	1.00
Ca574	Ca	0.59375	0.57317	0.10295	0.00000	Uiso	1.00

Ca575	Ca	0.65625	0.75347	0.10295	0.00000	Uiso	1.00
Ca576	Ca	0.71875	0.57317	0.10295	0.00000	Uiso	1.00
Ca577	Ca	0.53125	0.54047	0.05305	0.00000	Uiso	1.00
Ca578	Ca	0.59375	0.26717	0.05305	0.00000	Uiso	1.00
Ca579	Ca	0.65625	0.54047	0.05305	0.00000	Uiso	1.00
Ca580	Ca	0.71875	0.26717	0.05305	0.00000	Uiso	1.00
Ca581	Ca	0.53125	0.29997	0.10295	0.00000	Uiso	1.00
Ca582	Ca	0.59375	0.02667	0.10295	0.00000	Uiso	1.00
Ca583	Ca	0.65625	0.29997	0.10295	0.00000	Uiso	1.00
Ca584	Ca	0.71875	0.02667	0.10295	0.00000	Uiso	1.00
Ca585	Ca	0.59375	0.87372	0.07800	0.00000	Uiso	0.25
Ca586	Ca	0.71875	0.87372	0.07800	0.00000	Uiso	0.25
Ca587	Ca	0.53125	0.14692	0.07800	0.00000	Uiso	0.25
Ca588	Ca	0.59375	0.42022	0.07800	0.00000	Uiso	0.25
Ca589	Ca	0.65625	0.14692	0.07800	0.00000	Uiso	0.25
Ca590	Ca	0.71875	0.42022	0.07800	0.00000	Uiso	0.25
Ca591	Ca	0.53125	0.65348	0.03120	0.00000	Uiso	0.25
Ca592	Ca	0.65625	0.65348	0.03120	0.00000	Uiso	0.25
O593	O	0.61322	0.80674	0.06241	0.00000	Uiso	1.00
O594	O	0.70193	0.35324	0.06241	0.00000	Uiso	1.00
O595	O	0.57692	0.48710	0.09359	0.00000	Uiso	1.00
O596	O	0.73822	0.94060	0.09359	0.00000	Uiso	1.00
O597	O	0.51177	0.08004	0.06241	0.00000	Uiso	1.00
O598	O	0.61322	0.35324	0.06241	0.00000	Uiso	1.00
O599	O	0.67307	0.08004	0.06241	0.00000	Uiso	1.00
O600	O	0.70193	0.80674	0.06241	0.00000	Uiso	1.00
O601	O	0.54807	0.21390	0.09359	0.00000	Uiso	1.00
O602	O	0.57692	0.94060	0.09359	0.00000	Uiso	1.00
O603	O	0.63677	0.21390	0.09359	0.00000	Uiso	1.00
O604	O	0.73822	0.48710	0.09359	0.00000	Uiso	1.00
O605	O	0.53125	0.25486	0.12120	0.00000	Uiso	1.00
O606	O	0.59375	0.98166	0.12120	0.00000	Uiso	1.00
O607	O	0.65625	0.70836	0.12120	0.00000	Uiso	1.00
O608	O	0.71875	0.98166	0.12120	0.00000	Uiso	1.00
O609	O	0.53125	0.58549	0.03480	0.00000	Uiso	1.00
O610	O	0.59375	0.76579	0.03480	0.00000	Uiso	1.00
O611	O	0.65625	0.03899	0.03480	0.00000	Uiso	1.00
O612	O	0.71875	0.76579	0.03480	0.00000	Uiso	1.00
O613	O	0.53125	0.70836	0.12120	0.00000	Uiso	1.00
O614	O	0.59375	0.52816	0.12120	0.00000	Uiso	1.00
O615	O	0.65625	0.25486	0.12120	0.00000	Uiso	1.00
O616	O	0.71875	0.52816	0.12120	0.00000	Uiso	1.00
O617	O	0.53125	0.03899	0.03480	0.00000	Uiso	1.00
O618	O	0.59375	0.31229	0.03480	0.00000	Uiso	1.00
O619	O	0.65625	0.58549	0.03480	0.00000	Uiso	1.00
O620	O	0.71875	0.31229	0.03480	0.00000	Uiso	1.00
O621	O	0.53125	0.03422	0.07800	0.00000	Uiso	1.00
O622	O	0.53125	0.25962	0.07800	0.00000	Uiso	1.00
O623	O	0.53125	0.58072	0.07800	0.00000	Uiso	1.00
O624	O	0.53125	0.71312	0.07800	0.00000	Uiso	1.00
O625	O	0.59375	0.30752	0.07800	0.00000	Uiso	1.00
O626	O	0.59375	0.53292	0.07800	0.00000	Uiso	1.00
O627	O	0.59375	0.76102	0.07800	0.00000	Uiso	1.00
O628	O	0.59375	0.98642	0.07800	0.00000	Uiso	1.00
O629	O	0.65625	0.03422	0.07800	0.00000	Uiso	1.00
O630	O	0.65625	0.25962	0.07800	0.00000	Uiso	1.00
O631	O	0.65625	0.58072	0.07800	0.00000	Uiso	1.00
O632	O	0.65625	0.71312	0.07800	0.00000	Uiso	1.00

O633	O	0.71875	0.30752	0.07800	0.00000	Uiso	1.00
O634	O	0.71875	0.53292	0.07800	0.00000	Uiso	1.00
O635	O	0.71875	0.76102	0.07800	0.00000	Uiso	1.00
O636	O	0.71875	0.98642	0.07800	0.00000	Uiso	1.00
O637	O	0.53125	0.33535	0.05776	0.00000	Uiso	1.00
O638	O	0.53125	0.78885	0.05776	0.00000	Uiso	1.00
O639	O	0.59375	0.06205	0.05776	0.00000	Uiso	1.00
O640	O	0.59375	0.60855	0.05776	0.00000	Uiso	1.00
O641	O	0.65625	0.50509	0.09823	0.00000	Uiso	1.00
O642	O	0.65625	0.95859	0.09823	0.00000	Uiso	1.00
O643	O	0.71875	0.23179	0.09823	0.00000	Uiso	1.00
O644	O	0.71875	0.68529	0.09823	0.00000	Uiso	1.00
O645	O	0.51177	0.62654	0.06241	0.00000	Uiso	1.00
O646	O	0.67307	0.62654	0.06241	0.00000	Uiso	1.00
O647	O	0.54807	0.66740	0.09359	0.00000	Uiso	1.00
O648	O	0.63677	0.66740	0.09359	0.00000	Uiso	1.00
O649	O	0.53125	0.44912	0.07800	0.00000	Uiso	1.00
O650	O	0.53125	0.90262	0.07800	0.00000	Uiso	1.00
O651	O	0.59375	0.17592	0.07800	0.00000	Uiso	1.00
O652	O	0.65625	0.39122	0.07800	0.00000	Uiso	1.00
O653	O	0.65625	0.84472	0.07800	0.00000	Uiso	1.00
O654	O	0.71875	0.11802	0.07800	0.00000	Uiso	1.00
O655	O	0.53125	0.81266	0.00874	0.00000	Uiso	1.00
O656	O	0.65625	0.93468	0.14726	0.00000	Uiso	1.00
O657	O	0.53125	0.35916	0.00874	0.00000	Uiso	1.00
O658	O	0.59375	0.08596	0.00874	0.00000	Uiso	1.00
O659	O	0.65625	0.48118	0.14726	0.00000	Uiso	1.00
O660	O	0.71875	0.20798	0.14726	0.00000	Uiso	1.00
O661	O	0.53125	0.39122	0.07800	0.00000	Uiso	0.25
O662	O	0.53125	0.84472	0.07800	0.00000	Uiso	0.25
O663	O	0.59375	0.11802	0.07800	0.00000	Uiso	0.25
O664	O	0.65625	0.44912	0.07800	0.00000	Uiso	0.25
O665	O	0.65625	0.90262	0.07800	0.00000	Uiso	0.25
O666	O	0.71875	0.17592	0.07800	0.00000	Uiso	0.25
O667	O	0.59375	0.63215	0.01155	0.00000	Uiso	1.00
O668	O	0.71875	0.63215	0.01155	0.00000	Uiso	1.00
O669	O	0.59375	0.66179	0.14445	0.00000	Uiso	1.00
O670	O	0.71875	0.66179	0.14445	0.00000	Uiso	1.00
O671	O	0.53125	0.48176	0.14203	0.00000	Uiso	1.00
O672	O	0.53125	0.93526	0.14203	0.00000	Uiso	1.00
O673	O	0.59375	0.20856	0.14203	0.00000	Uiso	1.00
O674	O	0.65625	0.35858	0.01396	0.00000	Uiso	1.00
O675	O	0.65625	0.81208	0.01396	0.00000	Uiso	1.00
O676	O	0.71875	0.08538	0.01396	0.00000	Uiso	1.00
O677	O	0.53125	0.15287	0.02409	0.00000	Uiso	0.25
O678	O	0.59375	0.42617	0.02409	0.00000	Uiso	0.25
O679	O	0.59375	0.87967	0.02409	0.00000	Uiso	0.25
O680	O	0.65625	0.15130	0.03832	0.00000	Uiso	0.25
O681	O	0.71875	0.42460	0.03832	0.00000	Uiso	0.25
O682	O	0.71875	0.87810	0.03832	0.00000	Uiso	0.25
O683	O	0.53125	0.14254	0.11768	0.00000	Uiso	0.25
O684	O	0.59375	0.41584	0.11768	0.00000	Uiso	0.25
O685	O	0.59375	0.86934	0.11768	0.00000	Uiso	0.25
O686	O	0.65625	0.14098	0.13191	0.00000	Uiso	0.25
O687	O	0.71875	0.41428	0.13191	0.00000	Uiso	0.25
O688	O	0.71875	0.86778	0.13191	0.00000	Uiso	0.25
Si689	Si	0.54075	0.05333	0.05795	0.00000	Uiso	1.00
Si690	Si	0.54075	0.59983	0.05795	0.00000	Uiso	1.00

Si691	Si	0.58425	0.32653	0.05795	0.00000	Uiso	1.00
Si692	Si	0.58425	0.78003	0.05795	0.00000	Uiso	1.00
Si693	Si	0.66575	0.24061	0.09805	0.00000	Uiso	1.00
Si694	Si	0.66575	0.69411	0.09805	0.00000	Uiso	1.00
Si695	Si	0.70925	0.51381	0.09805	0.00000	Uiso	1.00
Si696	Si	0.70925	0.96731	0.09805	0.00000	Uiso	1.00
Si697	Si	0.53125	0.47972	0.07800	0.00000	Uiso	1.00
Si698	Si	0.53125	0.93322	0.07800	0.00000	Uiso	1.00
Si699	Si	0.59375	0.20652	0.07800	0.00000	Uiso	1.00
Si700	Si	0.65625	0.36062	0.07800	0.00000	Uiso	1.00
Si701	Si	0.65625	0.81412	0.07800	0.00000	Uiso	1.00
Si702	Si	0.71875	0.08742	0.07800	0.00000	Uiso	1.00
Si703	Si	0.64582	0.05357	0.05849	0.00000	Uiso	1.00
Si704	Si	0.64582	0.60007	0.05849	0.00000	Uiso	1.00
Si705	Si	0.72917	0.32677	0.05849	0.00000	Uiso	1.00
Si706	Si	0.72917	0.78037	0.05849	0.00000	Uiso	1.00
Si707	Si	0.52082	0.24027	0.09750	0.00000	Uiso	1.00
Si708	Si	0.52082	0.69387	0.09750	0.00000	Uiso	1.00
Si709	Si	0.60417	0.51357	0.09750	0.00000	Uiso	1.00
Si710	Si	0.60417	0.96707	0.09750	0.00000	Uiso	1.00
Ca711	Ca	0.65625	0.98365	0.14664	0.00000	Uiso	1.00
Ca712	Ca	0.71875	0.71035	0.14664	0.00000	Uiso	1.00
Ca713	Ca	0.53125	0.98365	0.14664	0.00000	Uiso	1.00
Ca714	Ca	0.59375	0.71035	0.14664	0.00000	Uiso	1.00
Ca715	Ca	0.65625	0.76379	0.00936	0.00000	Uiso	1.00
Ca716	Ca	0.71875	0.58349	0.00936	0.00000	Uiso	1.00
Ca717	Ca	0.53125	0.76379	0.00936	0.00000	Uiso	1.00
Ca718	Ca	0.59375	0.58349	0.00936	0.00000	Uiso	1.00
Ca719	Ca	0.65625	0.53015	0.14664	0.00000	Uiso	1.00
Ca720	Ca	0.71875	0.25685	0.14664	0.00000	Uiso	1.00
Ca721	Ca	0.53125	0.53015	0.14664	0.00000	Uiso	1.00
Ca722	Ca	0.59375	0.25685	0.14664	0.00000	Uiso	1.00
Ca723	Ca	0.65625	0.31029	0.00936	0.00000	Uiso	1.00
Ca724	Ca	0.71875	0.03699	0.00936	0.00000	Uiso	1.00
Ca725	Ca	0.53125	0.31029	0.00936	0.00000	Uiso	1.00
Ca726	Ca	0.59375	0.03699	0.00936	0.00000	Uiso	1.00
Ca727	Ca	0.71875	0.86340	0.17159	0.00000	Uiso	0.25
Ca728	Ca	0.59375	0.86340	0.17159	0.00000	Uiso	0.25
Ca729	Ca	0.65625	0.13660	0.17159	0.00000	Uiso	0.25
Ca730	Ca	0.71875	0.40990	0.17159	0.00000	Uiso	0.25
Ca731	Ca	0.53125	0.13660	0.17159	0.00000	Uiso	0.25
Ca732	Ca	0.59375	0.40990	0.17159	0.00000	Uiso	0.25
Ca733	Ca	0.65625	0.64316	0.12480	0.00000	Uiso	0.25
Ca734	Ca	0.53125	0.64316	0.12480	0.00000	Uiso	0.25
O735	O	0.73823	0.79642	0.15600	0.00000	Uiso	1.00
O736	O	0.57692	0.34292	0.15600	0.00000	Uiso	1.00
O737	O	0.70193	0.49742	0.00000	0.00000	Uiso	1.00
O738	O	0.61322	0.95092	0.00000	0.00000	Uiso	1.00
O739	O	0.63677	0.06972	0.15600	0.00000	Uiso	1.00
O740	O	0.73823	0.34292	0.15600	0.00000	Uiso	1.00
O741	O	0.54807	0.06972	0.15600	0.00000	Uiso	1.00
O742	O	0.57692	0.79642	0.15600	0.00000	Uiso	1.00
O743	O	0.67307	0.22422	0.00000	0.00000	Uiso	1.00
O744	O	0.70193	0.95092	0.00000	0.00000	Uiso	1.00
O745	O	0.51177	0.22422	0.00000	0.00000	Uiso	1.00
O746	O	0.61322	0.49742	0.00000	0.00000	Uiso	1.00
O747	O	0.65625	0.26518	0.02761	0.00000	Uiso	1.00
O748	O	0.71875	0.99198	0.02761	0.00000	Uiso	1.00

O749	O	0.53125	0.71868	0.02761	0.00000	Uiso	1.00
O750	O	0.59375	0.99198	0.02761	0.00000	Uiso	1.00
O751	O	0.65625	0.57516	0.12839	0.00000	Uiso	1.00
O752	O	0.71875	0.75546	0.12839	0.00000	Uiso	1.00
O753	O	0.53125	0.02866	0.12839	0.00000	Uiso	1.00
O754	O	0.59375	0.75546	0.12839	0.00000	Uiso	1.00
O755	O	0.65625	0.71868	0.02761	0.00000	Uiso	1.00
O756	O	0.71875	0.53848	0.02761	0.00000	Uiso	1.00
O757	O	0.53125	0.26518	0.02761	0.00000	Uiso	1.00
O758	O	0.59375	0.53848	0.02761	0.00000	Uiso	1.00
O759	O	0.65625	0.02866	0.12839	0.00000	Uiso	1.00
O760	O	0.71875	0.30196	0.12839	0.00000	Uiso	1.00
O761	O	0.53125	0.57516	0.12839	0.00000	Uiso	1.00
O762	O	0.59375	0.30196	0.12839	0.00000	Uiso	1.00
O763	O	0.65625	0.02390	0.17159	0.00000	Uiso	1.00
O764	O	0.65625	0.24930	0.17159	0.00000	Uiso	1.00
O765	O	0.65625	0.57040	0.17159	0.00000	Uiso	1.00
O766	O	0.65625	0.70280	0.17159	0.00000	Uiso	1.00
O767	O	0.71875	0.29720	0.17159	0.00000	Uiso	1.00
O768	O	0.71875	0.52260	0.17159	0.00000	Uiso	1.00
O769	O	0.71875	0.75070	0.17159	0.00000	Uiso	1.00
O770	O	0.71875	0.97610	0.17159	0.00000	Uiso	1.00
O771	O	0.53125	0.02390	0.17159	0.00000	Uiso	1.00
O772	O	0.53125	0.24930	0.17159	0.00000	Uiso	1.00
O773	O	0.53125	0.57040	0.17159	0.00000	Uiso	1.00
O774	O	0.53125	0.70280	0.17159	0.00000	Uiso	1.00
O775	O	0.59375	0.29720	0.17159	0.00000	Uiso	1.00
O776	O	0.59375	0.52260	0.17159	0.00000	Uiso	1.00
O777	O	0.59375	0.75070	0.17159	0.00000	Uiso	1.00
O778	O	0.59375	0.97610	0.17159	0.00000	Uiso	1.00
O779	O	0.65625	0.32503	0.15136	0.00000	Uiso	1.00
O780	O	0.65625	0.77853	0.15136	0.00000	Uiso	1.00
O781	O	0.71875	0.05173	0.15136	0.00000	Uiso	1.00
O782	O	0.71875	0.59823	0.15136	0.00000	Uiso	1.00
O783	O	0.53125	0.51541	0.00464	0.00000	Uiso	1.00
O784	O	0.53125	0.96891	0.00464	0.00000	Uiso	1.00
O785	O	0.59375	0.24211	0.00464	0.00000	Uiso	1.00
O786	O	0.59375	0.69561	0.00464	0.00000	Uiso	1.00
O787	O	0.63677	0.61622	0.15600	0.00000	Uiso	1.00
O788	O	0.54807	0.61622	0.15600	0.00000	Uiso	1.00
O789	O	0.67307	0.67772	0.00000	0.00000	Uiso	1.00
O790	O	0.51177	0.67772	0.00000	0.00000	Uiso	1.00
O791	O	0.65625	0.43880	0.17159	0.00000	Uiso	1.00
O792	O	0.65625	0.89230	0.17159	0.00000	Uiso	1.00
O793	O	0.71875	0.16560	0.17159	0.00000	Uiso	1.00
O794	O	0.53125	0.38090	0.17159	0.00000	Uiso	1.00
O795	O	0.53125	0.83440	0.17159	0.00000	Uiso	1.00
O796	O	0.59375	0.10770	0.17159	0.00000	Uiso	1.00
O797	O	0.65625	0.80234	0.10233	0.00000	Uiso	1.00
O798	O	0.53125	0.94500	0.05367	0.00000	Uiso	1.00
O799	O	0.65625	0.34884	0.10233	0.00000	Uiso	1.00
O800	O	0.71875	0.07564	0.10233	0.00000	Uiso	1.00
O801	O	0.53125	0.49150	0.05367	0.00000	Uiso	1.00
O802	O	0.59375	0.21830	0.05367	0.00000	Uiso	1.00
O803	O	0.65625	0.38090	0.17159	0.00000	Uiso	0.25
O804	O	0.65625	0.83440	0.17159	0.00000	Uiso	0.25
O805	O	0.71875	0.10770	0.17159	0.00000	Uiso	0.25
O806	O	0.53125	0.43880	0.17159	0.00000	Uiso	0.25

O807	O	0.53125	0.89230	0.17159	0.00000	Uiso	0.25
O808	O	0.59375	0.16560	0.17159	0.00000	Uiso	0.25
O809	O	0.71875	0.62183	0.10514	0.00000	Uiso	1.00
O810	O	0.59375	0.62183	0.10514	0.00000	Uiso	1.00
O811	O	0.71875	0.67211	0.05086	0.00000	Uiso	1.00
O812	O	0.59375	0.67211	0.05086	0.00000	Uiso	1.00
O813	O	0.65625	0.49208	0.04844	0.00000	Uiso	1.00
O814	O	0.65625	0.94558	0.04844	0.00000	Uiso	1.00
O815	O	0.71875	0.21888	0.04844	0.00000	Uiso	1.00
O816	O	0.53125	0.34826	0.10756	0.00000	Uiso	1.00
O817	O	0.53125	0.80176	0.10756	0.00000	Uiso	1.00
O818	O	0.59375	0.07506	0.10756	0.00000	Uiso	1.00
O819	O	0.65625	0.14254	0.11768	0.00000	Uiso	0.25
O820	O	0.71875	0.41584	0.11768	0.00000	Uiso	0.25
O821	O	0.71875	0.86934	0.11768	0.00000	Uiso	0.25
O822	O	0.53125	0.14098	0.13191	0.00000	Uiso	0.25
O823	O	0.59375	0.41428	0.13191	0.00000	Uiso	0.25
O824	O	0.59375	0.86778	0.13191	0.00000	Uiso	0.25
O825	O	0.65625	0.15287	0.02409	0.00000	Uiso	0.25
O826	O	0.71875	0.42617	0.02409	0.00000	Uiso	0.25
O827	O	0.71875	0.87967	0.02409	0.00000	Uiso	0.25
O828	O	0.53125	0.15130	0.03832	0.00000	Uiso	0.25
O829	O	0.59375	0.42460	0.03832	0.00000	Uiso	0.25
O830	O	0.59375	0.87810	0.03832	0.00000	Uiso	0.25
Si831	Si	0.66575	0.04301	0.15154	0.00000	Uiso	1.00
Si832	Si	0.66575	0.58951	0.15154	0.00000	Uiso	1.00
Si833	Si	0.70925	0.31621	0.15154	0.00000	Uiso	1.00
Si834	Si	0.70925	0.76971	0.15154	0.00000	Uiso	1.00
Si835	Si	0.54075	0.25093	0.00445	0.00000	Uiso	1.00
Si836	Si	0.54075	0.70443	0.00445	0.00000	Uiso	1.00
Si837	Si	0.58425	0.52413	0.00445	0.00000	Uiso	1.00
Si838	Si	0.58425	0.97763	0.00445	0.00000	Uiso	1.00
Si839	Si	0.65625	0.46940	0.17159	0.00000	Uiso	1.00
Si840	Si	0.65625	0.92290	0.17159	0.00000	Uiso	1.00
Si841	Si	0.71875	0.19620	0.17159	0.00000	Uiso	1.00
Si842	Si	0.53125	0.35030	0.17159	0.00000	Uiso	1.00
Si843	Si	0.53125	0.80380	0.17159	0.00000	Uiso	1.00
Si844	Si	0.59375	0.07710	0.17159	0.00000	Uiso	1.00
Si845	Si	0.52082	0.04325	0.15209	0.00000	Uiso	1.00
Si846	Si	0.52082	0.58975	0.15209	0.00000	Uiso	1.00
Si847	Si	0.60417	0.31645	0.15209	0.00000	Uiso	1.00
Si848	Si	0.60417	0.77005	0.15209	0.00000	Uiso	1.00
Si849	Si	0.64582	0.25059	0.00391	0.00000	Uiso	1.00
Si850	Si	0.64582	0.70419	0.00391	0.00000	Uiso	1.00
Si851	Si	0.72918	0.52389	0.00391	0.00000	Uiso	1.00
Si852	Si	0.72918	0.97739	0.00391	0.00000	Uiso	1.00
Ca853	Ca	0.78125	0.99397	0.05305	0.00000	Uiso	1.00
Ca854	Ca	0.84375	0.72067	0.05305	0.00000	Uiso	1.00
Ca855	Ca	0.90625	0.99397	0.05305	0.00000	Uiso	1.00
Ca856	Ca	0.96875	0.72067	0.05305	0.00000	Uiso	1.00
Ca857	Ca	0.78125	0.75347	0.10295	0.00000	Uiso	1.00
Ca858	Ca	0.84375	0.57317	0.10295	0.00000	Uiso	1.00
Ca859	Ca	0.90625	0.75347	0.10295	0.00000	Uiso	1.00
Ca860	Ca	0.96875	0.57317	0.10295	0.00000	Uiso	1.00
Ca861	Ca	0.78125	0.54047	0.05305	0.00000	Uiso	1.00
Ca862	Ca	0.84375	0.26717	0.05305	0.00000	Uiso	1.00
Ca863	Ca	0.90625	0.54047	0.05305	0.00000	Uiso	1.00
Ca864	Ca	0.96875	0.26717	0.05305	0.00000	Uiso	1.00

Ca865	Ca	0.78125	0.29997	0.10295	0.00000	Uiso	1.00
Ca866	Ca	0.84375	0.02667	0.10295	0.00000	Uiso	1.00
Ca867	Ca	0.90625	0.29997	0.10295	0.00000	Uiso	1.00
Ca868	Ca	0.96875	0.02667	0.10295	0.00000	Uiso	1.00
Ca869	Ca	0.84375	0.87372	0.07800	0.00000	Uiso	0.25
Ca870	Ca	0.96875	0.87372	0.07800	0.00000	Uiso	0.25
Ca871	Ca	0.78125	0.14692	0.07800	0.00000	Uiso	0.25
Ca872	Ca	0.84375	0.42022	0.07800	0.00000	Uiso	0.25
Ca873	Ca	0.90625	0.14692	0.07800	0.00000	Uiso	0.25
Ca874	Ca	0.96875	0.42022	0.07800	0.00000	Uiso	0.25
Ca875	Ca	0.78125	0.65348	0.03120	0.00000	Uiso	0.25
Ca876	Ca	0.90625	0.65348	0.03120	0.00000	Uiso	0.25
O877	O	0.86322	0.80674	0.06241	0.00000	Uiso	1.00
O878	O	0.95192	0.35324	0.06241	0.00000	Uiso	1.00
O879	O	0.82692	0.48710	0.09359	0.00000	Uiso	1.00
O880	O	0.98822	0.94060	0.09359	0.00000	Uiso	1.00
O881	O	0.76177	0.08004	0.06241	0.00000	Uiso	1.00
O882	O	0.86322	0.35324	0.06241	0.00000	Uiso	1.00
O883	O	0.92307	0.08004	0.06241	0.00000	Uiso	1.00
O884	O	0.95192	0.80674	0.06241	0.00000	Uiso	1.00
O885	O	0.79807	0.21390	0.09359	0.00000	Uiso	1.00
O886	O	0.82692	0.94060	0.09359	0.00000	Uiso	1.00
O887	O	0.88677	0.21390	0.09359	0.00000	Uiso	1.00
O888	O	0.98822	0.48710	0.09359	0.00000	Uiso	1.00
O889	O	0.78125	0.25486	0.12120	0.00000	Uiso	1.00
O890	O	0.84375	0.98166	0.12120	0.00000	Uiso	1.00
O891	O	0.90625	0.70836	0.12120	0.00000	Uiso	1.00
O892	O	0.96875	0.98166	0.12120	0.00000	Uiso	1.00
O893	O	0.78125	0.58549	0.03480	0.00000	Uiso	1.00
O894	O	0.84375	0.76579	0.03480	0.00000	Uiso	1.00
O895	O	0.90625	0.03899	0.03480	0.00000	Uiso	1.00
O896	O	0.96875	0.76579	0.03480	0.00000	Uiso	1.00
O897	O	0.78125	0.70836	0.12120	0.00000	Uiso	1.00
O898	O	0.84375	0.52816	0.12120	0.00000	Uiso	1.00
O899	O	0.90625	0.25486	0.12120	0.00000	Uiso	1.00
O900	O	0.96875	0.52816	0.12120	0.00000	Uiso	1.00
O901	O	0.78125	0.03899	0.03480	0.00000	Uiso	1.00
O902	O	0.84375	0.31229	0.03480	0.00000	Uiso	1.00
O903	O	0.90625	0.58549	0.03480	0.00000	Uiso	1.00
O904	O	0.96875	0.31229	0.03480	0.00000	Uiso	1.00
O905	O	0.78125	0.03422	0.07800	0.00000	Uiso	1.00
O906	O	0.78125	0.25962	0.07800	0.00000	Uiso	1.00
O907	O	0.78125	0.58072	0.07800	0.00000	Uiso	1.00
O908	O	0.78125	0.71312	0.07800	0.00000	Uiso	1.00
O909	O	0.84375	0.30752	0.07800	0.00000	Uiso	1.00
O910	O	0.84375	0.53292	0.07800	0.00000	Uiso	1.00
O911	O	0.84375	0.76102	0.07800	0.00000	Uiso	1.00
O912	O	0.84375	0.98642	0.07800	0.00000	Uiso	1.00
O913	O	0.90625	0.03422	0.07800	0.00000	Uiso	1.00
O914	O	0.90625	0.25962	0.07800	0.00000	Uiso	1.00
O915	O	0.90625	0.58072	0.07800	0.00000	Uiso	1.00
O916	O	0.90625	0.71312	0.07800	0.00000	Uiso	1.00
O917	O	0.96875	0.30752	0.07800	0.00000	Uiso	1.00
O918	O	0.96875	0.53292	0.07800	0.00000	Uiso	1.00
O919	O	0.96875	0.76102	0.07800	0.00000	Uiso	1.00
O920	O	0.96875	0.98642	0.07800	0.00000	Uiso	1.00
O921	O	0.78125	0.33535	0.05776	0.00000	Uiso	1.00
O922	O	0.78125	0.78885	0.05776	0.00000	Uiso	1.00

O923	O	0.84375	0.06205	0.05776	0.00000	Uiso	1.00
O924	O	0.84375	0.60855	0.05776	0.00000	Uiso	1.00
O925	O	0.90625	0.50509	0.09823	0.00000	Uiso	1.00
O926	O	0.90625	0.95859	0.09823	0.00000	Uiso	1.00
O927	O	0.96875	0.23179	0.09823	0.00000	Uiso	1.00
O928	O	0.96875	0.68529	0.09823	0.00000	Uiso	1.00
O929	O	0.76177	0.62654	0.06241	0.00000	Uiso	1.00
O930	O	0.92307	0.62654	0.06241	0.00000	Uiso	1.00
O931	O	0.79807	0.66740	0.09359	0.00000	Uiso	1.00
O932	O	0.88677	0.66740	0.09359	0.00000	Uiso	1.00
O933	O	0.78125	0.44912	0.07800	0.00000	Uiso	1.00
O934	O	0.78125	0.90262	0.07800	0.00000	Uiso	1.00
O935	O	0.84375	0.17592	0.07800	0.00000	Uiso	1.00
O936	O	0.90625	0.39122	0.07800	0.00000	Uiso	1.00
O937	O	0.90625	0.84472	0.07800	0.00000	Uiso	1.00
O938	O	0.96875	0.11802	0.07800	0.00000	Uiso	1.00
O939	O	0.78125	0.81266	0.00874	0.00000	Uiso	1.00
O940	O	0.90625	0.93468	0.14726	0.00000	Uiso	1.00
O941	O	0.78125	0.35916	0.00874	0.00000	Uiso	1.00
O942	O	0.84375	0.08596	0.00874	0.00000	Uiso	1.00
O943	O	0.90625	0.48118	0.14726	0.00000	Uiso	1.00
O944	O	0.96875	0.20798	0.14726	0.00000	Uiso	1.00
O945	O	0.78125	0.39122	0.07800	0.00000	Uiso	0.25
O946	O	0.78125	0.84472	0.07800	0.00000	Uiso	0.25
O947	O	0.84375	0.11802	0.07800	0.00000	Uiso	0.25
O948	O	0.90625	0.44912	0.07800	0.00000	Uiso	0.25
O949	O	0.90625	0.90262	0.07800	0.00000	Uiso	0.25
O950	O	0.96875	0.17592	0.07800	0.00000	Uiso	0.25
O951	O	0.84375	0.63215	0.01155	0.00000	Uiso	1.00
O952	O	0.96875	0.63215	0.01155	0.00000	Uiso	1.00
O953	O	0.84375	0.66179	0.14445	0.00000	Uiso	1.00
O954	O	0.96875	0.66179	0.14445	0.00000	Uiso	1.00
O955	O	0.78125	0.48176	0.14203	0.00000	Uiso	1.00
O956	O	0.78125	0.93526	0.14203	0.00000	Uiso	1.00
O957	O	0.84375	0.20856	0.14203	0.00000	Uiso	1.00
O958	O	0.90625	0.35858	0.01396	0.00000	Uiso	1.00
O959	O	0.90625	0.81208	0.01396	0.00000	Uiso	1.00
O960	O	0.96875	0.08538	0.01396	0.00000	Uiso	1.00
O961	O	0.78125	0.15287	0.02409	0.00000	Uiso	0.25
O962	O	0.84375	0.42617	0.02409	0.00000	Uiso	0.25
O963	O	0.84375	0.87967	0.02409	0.00000	Uiso	0.25
O964	O	0.90625	0.15130	0.03832	0.00000	Uiso	0.25
O965	O	0.96875	0.42460	0.03832	0.00000	Uiso	0.25
O966	O	0.96875	0.87810	0.03832	0.00000	Uiso	0.25
O967	O	0.78125	0.14254	0.11768	0.00000	Uiso	0.25
O968	O	0.84375	0.41584	0.11768	0.00000	Uiso	0.25
O969	O	0.84375	0.86934	0.11768	0.00000	Uiso	0.25
O970	O	0.90625	0.14098	0.13191	0.00000	Uiso	0.25
O971	O	0.96875	0.41428	0.13191	0.00000	Uiso	0.25
O972	O	0.96875	0.86778	0.13191	0.00000	Uiso	0.25
Si973	Si	0.79075	0.05333	0.05795	0.00000	Uiso	1.00
Si974	Si	0.79075	0.59983	0.05795	0.00000	Uiso	1.00
Si975	Si	0.83425	0.32653	0.05795	0.00000	Uiso	1.00
Si976	Si	0.83425	0.78003	0.05795	0.00000	Uiso	1.00
Si977	Si	0.91575	0.24061	0.09805	0.00000	Uiso	1.00
Si978	Si	0.91575	0.69411	0.09805	0.00000	Uiso	1.00
Si979	Si	0.95925	0.51381	0.09805	0.00000	Uiso	1.00
Si980	Si	0.95925	0.96731	0.09805	0.00000	Uiso	1.00

Si981	Si	0.78125	0.47972	0.07800	0.00000	Uiso	1.00
Si982	Si	0.78125	0.93322	0.07800	0.00000	Uiso	1.00
Si983	Si	0.84375	0.20652	0.07800	0.00000	Uiso	1.00
Si984	Si	0.90625	0.36062	0.07800	0.00000	Uiso	1.00
Si985	Si	0.90625	0.81412	0.07800	0.00000	Uiso	1.00
Si986	Si	0.96875	0.08742	0.07800	0.00000	Uiso	1.00
Si987	Si	0.89582	0.05357	0.05849	0.00000	Uiso	1.00
Si988	Si	0.89582	0.60007	0.05849	0.00000	Uiso	1.00
Si989	Si	0.97917	0.32677	0.05849	0.00000	Uiso	1.00
Si990	Si	0.97917	0.78037	0.05849	0.00000	Uiso	1.00
Si991	Si	0.77082	0.24027	0.09750	0.00000	Uiso	1.00
Si992	Si	0.77082	0.69387	0.09750	0.00000	Uiso	1.00
Si993	Si	0.85417	0.51357	0.09750	0.00000	Uiso	1.00
Si994	Si	0.85417	0.96707	0.09750	0.00000	Uiso	1.00
Ca995	Ca	0.90625	0.98365	0.14664	0.00000	Uiso	1.00
Ca996	Ca	0.96875	0.71035	0.14664	0.00000	Uiso	1.00
Ca997	Ca	0.78125	0.98365	0.14664	0.00000	Uiso	1.00
Ca998	Ca	0.84375	0.71035	0.14664	0.00000	Uiso	1.00
Ca999	Ca	0.90625	0.76379	0.00936	0.00000	Uiso	1.00
Ca1000	Ca	0.96875	0.58349	0.00936	0.00000	Uiso	1.00
Ca1001	Ca	0.78125	0.76379	0.00936	0.00000	Uiso	1.00
Ca1002	Ca	0.84375	0.58349	0.00936	0.00000	Uiso	1.00
Ca1003	Ca	0.90625	0.53015	0.14664	0.00000	Uiso	1.00
Ca1004	Ca	0.96875	0.25685	0.14664	0.00000	Uiso	1.00
Ca1005	Ca	0.78125	0.53015	0.14664	0.00000	Uiso	1.00
Ca1006	Ca	0.84375	0.25685	0.14664	0.00000	Uiso	1.00
Ca1007	Ca	0.90625	0.31029	0.00936	0.00000	Uiso	1.00
Ca1008	Ca	0.96875	0.03699	0.00936	0.00000	Uiso	1.00
Ca1009	Ca	0.78125	0.31029	0.00936	0.00000	Uiso	1.00
Ca1010	Ca	0.84375	0.03699	0.00936	0.00000	Uiso	1.00
Ca1011	Ca	0.96875	0.86340	0.17159	0.00000	Uiso	0.25
Ca1012	Ca	0.84375	0.86340	0.17159	0.00000	Uiso	0.25
Ca1013	Ca	0.90625	0.13660	0.17159	0.00000	Uiso	0.25
Ca1014	Ca	0.96875	0.40990	0.17159	0.00000	Uiso	0.25
Ca1015	Ca	0.78125	0.13660	0.17159	0.00000	Uiso	0.25
Ca1016	Ca	0.84375	0.40990	0.17159	0.00000	Uiso	0.25
Ca1017	Ca	0.90625	0.64316	0.12480	0.00000	Uiso	0.25
Ca1018	Ca	0.78125	0.64316	0.12480	0.00000	Uiso	0.25
O1019	O	0.98822	0.79642	0.15600	0.00000	Uiso	1.00
O1020	O	0.82692	0.34292	0.15600	0.00000	Uiso	1.00
O1021	O	0.95192	0.49742	0.00000	0.00000	Uiso	1.00
O1022	O	0.86322	0.95092	0.00000	0.00000	Uiso	1.00
O1023	O	0.88677	0.06972	0.15600	0.00000	Uiso	1.00
O1024	O	0.98822	0.34292	0.15600	0.00000	Uiso	1.00
O1025	O	0.79807	0.06972	0.15600	0.00000	Uiso	1.00
O1026	O	0.82692	0.79642	0.15600	0.00000	Uiso	1.00
O1027	O	0.92307	0.22422	0.00000	0.00000	Uiso	1.00
O1028	O	0.95192	0.95092	0.00000	0.00000	Uiso	1.00
O1029	O	0.76177	0.22422	0.00000	0.00000	Uiso	1.00
O1030	O	0.86322	0.49742	0.00000	0.00000	Uiso	1.00
O1031	O	0.90625	0.26518	0.02761	0.00000	Uiso	1.00
O1032	O	0.96875	0.99198	0.02761	0.00000	Uiso	1.00
O1033	O	0.78125	0.71868	0.02761	0.00000	Uiso	1.00
O1034	O	0.84375	0.99198	0.02761	0.00000	Uiso	1.00
O1035	O	0.90625	0.57516	0.12839	0.00000	Uiso	1.00
O1036	O	0.96875	0.75546	0.12839	0.00000	Uiso	1.00
O1037	O	0.78125	0.02866	0.12839	0.00000	Uiso	1.00
O1038	O	0.84375	0.75546	0.12839	0.00000	Uiso	1.00

O1039	O	0.90625	0.71868	0.02761	0.00000	Uiso	1.00
O1040	O	0.96875	0.53848	0.02761	0.00000	Uiso	1.00
O1041	O	0.78125	0.26518	0.02761	0.00000	Uiso	1.00
O1042	O	0.84375	0.53848	0.02761	0.00000	Uiso	1.00
O1043	O	0.90625	0.02866	0.12839	0.00000	Uiso	1.00
O1044	O	0.96875	0.30196	0.12839	0.00000	Uiso	1.00
O1045	O	0.78125	0.57516	0.12839	0.00000	Uiso	1.00
O1046	O	0.84375	0.30196	0.12839	0.00000	Uiso	1.00
O1047	O	0.90625	0.02390	0.17159	0.00000	Uiso	1.00
O1048	O	0.90625	0.24930	0.17159	0.00000	Uiso	1.00
O1049	O	0.90625	0.57040	0.17159	0.00000	Uiso	1.00
O1050	O	0.90625	0.70280	0.17159	0.00000	Uiso	1.00
O1051	O	0.96875	0.29720	0.17159	0.00000	Uiso	1.00
O1052	O	0.96875	0.52260	0.17159	0.00000	Uiso	1.00
O1053	O	0.96875	0.75070	0.17159	0.00000	Uiso	1.00
O1054	O	0.96875	0.97610	0.17159	0.00000	Uiso	1.00
O1055	O	0.78125	0.02390	0.17159	0.00000	Uiso	1.00
O1056	O	0.78125	0.24930	0.17159	0.00000	Uiso	1.00
O1057	O	0.78125	0.57040	0.17159	0.00000	Uiso	1.00
O1058	O	0.78125	0.70280	0.17159	0.00000	Uiso	1.00
O1059	O	0.84375	0.29720	0.17159	0.00000	Uiso	1.00
O1060	O	0.84375	0.52260	0.17159	0.00000	Uiso	1.00
O1061	O	0.84375	0.75070	0.17159	0.00000	Uiso	1.00
O1062	O	0.84375	0.97610	0.17159	0.00000	Uiso	1.00
O1063	O	0.90625	0.32503	0.15136	0.00000	Uiso	1.00
O1064	O	0.90625	0.77853	0.15136	0.00000	Uiso	1.00
O1065	O	0.96875	0.05173	0.15136	0.00000	Uiso	1.00
O1066	O	0.96875	0.59823	0.15136	0.00000	Uiso	1.00
O1067	O	0.78125	0.51541	0.00464	0.00000	Uiso	1.00
O1068	O	0.78125	0.96891	0.00464	0.00000	Uiso	1.00
O1069	O	0.84375	0.24211	0.00464	0.00000	Uiso	1.00
O1070	O	0.84375	0.69561	0.00464	0.00000	Uiso	1.00
O1071	O	0.88677	0.61622	0.15600	0.00000	Uiso	1.00
O1072	O	0.79807	0.61622	0.15600	0.00000	Uiso	1.00
O1073	O	0.92307	0.67772	0.00000	0.00000	Uiso	1.00
O1074	O	0.76177	0.67772	0.00000	0.00000	Uiso	1.00
O1075	O	0.90625	0.43880	0.17159	0.00000	Uiso	1.00
O1076	O	0.90625	0.89230	0.17159	0.00000	Uiso	1.00
O1077	O	0.96875	0.16560	0.17159	0.00000	Uiso	1.00
O1078	O	0.78125	0.38090	0.17159	0.00000	Uiso	1.00
O1079	O	0.78125	0.83440	0.17159	0.00000	Uiso	1.00
O1080	O	0.84375	0.10770	0.17159	0.00000	Uiso	1.00
O1081	O	0.90625	0.80234	0.10233	0.00000	Uiso	1.00
O1082	O	0.78125	0.94500	0.05367	0.00000	Uiso	1.00
O1083	O	0.90625	0.34884	0.10233	0.00000	Uiso	1.00
O1084	O	0.96875	0.07564	0.10233	0.00000	Uiso	1.00
O1085	O	0.78125	0.49150	0.05367	0.00000	Uiso	1.00
O1086	O	0.84375	0.21830	0.05367	0.00000	Uiso	1.00
O1087	O	0.90625	0.38090	0.17159	0.00000	Uiso	0.25
O1088	O	0.90625	0.83440	0.17159	0.00000	Uiso	0.25
O1089	O	0.96875	0.10770	0.17159	0.00000	Uiso	0.25
O1090	O	0.78125	0.43880	0.17159	0.00000	Uiso	0.25
O1091	O	0.78125	0.89230	0.17159	0.00000	Uiso	0.25
O1092	O	0.84375	0.16560	0.17159	0.00000	Uiso	0.25
O1093	O	0.96875	0.62183	0.10514	0.00000	Uiso	1.00
O1094	O	0.84375	0.62183	0.10514	0.00000	Uiso	1.00
O1095	O	0.96875	0.67211	0.05086	0.00000	Uiso	1.00
O1096	O	0.84375	0.67211	0.05086	0.00000	Uiso	1.00

O1097	O	0.90625	0.49208	0.04844	0.00000	Uiso	1.00
O1098	O	0.90625	0.94558	0.04844	0.00000	Uiso	1.00
O1099	O	0.96875	0.21888	0.04844	0.00000	Uiso	1.00
O1100	O	0.78125	0.34826	0.10756	0.00000	Uiso	1.00
O1101	O	0.78125	0.80176	0.10756	0.00000	Uiso	1.00
O1102	O	0.84375	0.07506	0.10756	0.00000	Uiso	1.00
O1103	O	0.90625	0.14254	0.11768	0.00000	Uiso	0.25
O1104	O	0.96875	0.41584	0.11768	0.00000	Uiso	0.25
O1105	O	0.96875	0.86934	0.11768	0.00000	Uiso	0.25
O1106	O	0.78125	0.14098	0.13191	0.00000	Uiso	0.25
O1107	O	0.84375	0.41428	0.13191	0.00000	Uiso	0.25
O1108	O	0.84375	0.86778	0.13191	0.00000	Uiso	0.25
O1109	O	0.90625	0.15287	0.02409	0.00000	Uiso	0.25
O1110	O	0.96875	0.42617	0.02409	0.00000	Uiso	0.25
O1111	O	0.96875	0.87967	0.02409	0.00000	Uiso	0.25
O1112	O	0.78125	0.15130	0.03832	0.00000	Uiso	0.25
O1113	O	0.84375	0.42460	0.03832	0.00000	Uiso	0.25
O1114	O	0.84375	0.87810	0.03832	0.00000	Uiso	0.25
Si1115	Si	0.91575	0.04301	0.15154	0.00000	Uiso	1.00
Si1116	Si	0.91575	0.58951	0.15154	0.00000	Uiso	1.00
Si1117	Si	0.95925	0.31621	0.15154	0.00000	Uiso	1.00
Si1118	Si	0.95925	0.76971	0.15154	0.00000	Uiso	1.00
Si1119	Si	0.79075	0.25093	0.00445	0.00000	Uiso	1.00
Si1120	Si	0.79075	0.70443	0.00445	0.00000	Uiso	1.00
Si1121	Si	0.83425	0.52413	0.00445	0.00000	Uiso	1.00
Si1122	Si	0.83425	0.97763	0.00445	0.00000	Uiso	1.00
Si1123	Si	0.90625	0.46940	0.17159	0.00000	Uiso	1.00
Si1124	Si	0.90625	0.92290	0.17159	0.00000	Uiso	1.00
Si1125	Si	0.96875	0.19620	0.17159	0.00000	Uiso	1.00
Si1126	Si	0.78125	0.35030	0.17159	0.00000	Uiso	1.00
Si1127	Si	0.78125	0.80380	0.17159	0.00000	Uiso	1.00
Si1128	Si	0.84375	0.07710	0.17159	0.00000	Uiso	1.00
Si1129	Si	0.77082	0.04325	0.15209	0.00000	Uiso	1.00
Si1130	Si	0.77082	0.58975	0.15209	0.00000	Uiso	1.00
Si1131	Si	0.85417	0.31645	0.15209	0.00000	Uiso	1.00
Si1132	Si	0.85417	0.77005	0.15209	0.00000	Uiso	1.00
Si1133	Si	0.89582	0.25059	0.00391	0.00000	Uiso	1.00
Si1134	Si	0.89582	0.70419	0.00391	0.00000	Uiso	1.00
Si1135	Si	0.97917	0.52389	0.00391	0.00000	Uiso	1.00
Si1136	Si	0.97917	0.97739	0.00391	0.00000	Uiso	1.00

Model of the oligomer (O_{L1})

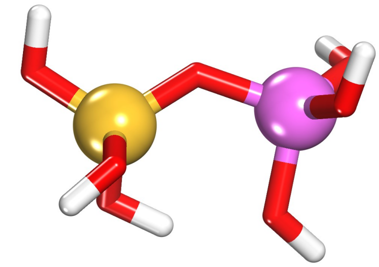

Si1	7.422047614	7.489088346	-1.721106714
O1	6.787890850	6.219884292	-2.566513640
O2	6.620564587	7.614313079	-0.209107387
O3	8.989723855	7.085626358	-1.404281579
Al1	8.083957151	10.451895455	-2.125863740
O4	6.957109299	11.475354676	-1.180614959
O5	9.117429279	9.880365603	-0.747979947
O6	7.191139126	8.930157739	-2.407572207
O7	8.811976938	11.287702940	-3.451744143
H1	6.026375388	6.477204757	-3.108044228
H2	6.790050497	6.832511627	0.340482492
H3	9.427878957	7.907665838	-1.086004531
H4	9.903343810	10.399242126	-0.528960493
H5	9.161790576	10.797130465	-4.206884946
H6	6.262366662	11.930988609	-1.674127400

Model of the oligomer (O_{L2})

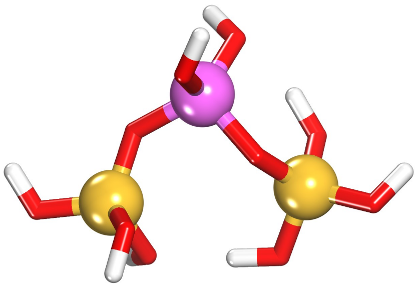

O1	3.730068665	6.928807048	-1.976213231
O2	5.032413017	1.886801518	-1.458615448
Si1	5.723081384	3.248515062	-0.805685374
Al1	4.726003711	6.171191333	-0.705312507
Si2	7.551264901	6.963601131	-1.623604139
O3	5.407564524	3.293988278	0.819517512
O4	4.065360226	5.829289870	0.874812151
O5	5.116345966	4.618000964	-1.493330414
O7	6.235891837	7.108061000	-0.726117960
O8	8.567104046	5.680736955	-1.293678180
O9	7.077010084	6.651996585	-3.218856642
O10	8.519669090	8.304868667	-1.546069730
H1	2.772684244	6.970861776	-1.854664308
H2	4.340946005	1.526897882	-0.883115851
H3	4.898620944	4.099310678	1.074022288
H4	4.191872508	6.438741814	1.613833524
H5	7.801135357	3.946863789	-1.191815354
H6	9.182313433	5.835884952	-0.559895444
H7	7.841315794	6.537484980	-3.805958305
H8	8.042196054	9.109844899	-1.292212631

Model of the oligomer (O_{C1})

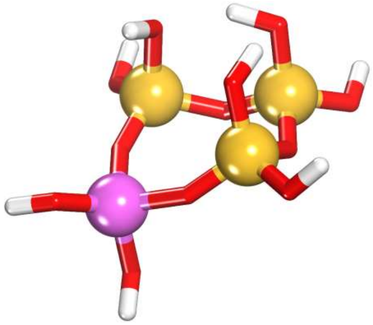

O1	3.711932239	20.844365034	3.870578901
O2	1.000705685	22.100889717	7.551547683
Si1	3.666854293	21.254353449	5.466045260
Si2	1.588729465	20.555830593	7.540482346
O3	3.721676596	21.099793261	11.170824218
O4	0.388305596	19.438541203	7.476445585
O5	2.535793715	20.237018299	6.233209198
O6	3.137723608	22.808570716	5.681011060
O7	5.098898908	20.930442379	6.099983448
O8	7.474808908	22.093632291	7.321025598
Si3	3.670165184	21.374765662	9.550472805
Al1	6.201250827	20.947201455	7.496433930
O9	6.535854882	19.200391765	7.714982655
O10	5.099749318	21.136939945	8.897633335
O11	3.025991328	22.899861113	9.241013547
O12	2.544161234	20.311681297	8.881871588
H1	3.000474078	21.225503833	3.335134926
H2	1.542776111	22.640418241	8.181073439
H3	2.929438812	21.347011259	11.670104207
H4	-0.361371465	19.598930221	8.068912775
H5	2.248584872	22.859379801	6.082103886
H6	8.145282154	22.192793480	8.008903085
H7	7.223032876	18.803606811	7.163056224
H8	3.656058416	23.487666312	8.791916514

Model of the oligomer (O_{C2})

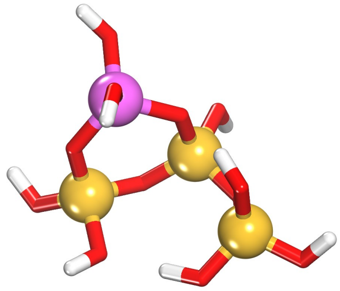

Al1	4.562685232	2.267284331	2.042312305
Si1	3.008854012	1.306335847	-0.414437607
O1	1.384764529	1.204852888	-0.696152619
O2	3.341425764	1.334880939	1.167164067
O3	3.733294441	0.046826094	-1.241023652
O4	3.650966411	2.636678993	-1.180298529
O5	3.915346283	3.742947467	2.828997671
Si2	5.075306804	3.366958138	-0.807452268
Si3	7.227660903	1.575122957	-1.739964552

O6	5.513757676	3.118451685	0.744579166
O7	4.835480944	5.035869454	-0.848582431
O8	6.247504293	2.936192966	-1.852451213
O9	7.843093907	1.537692718	-0.203127704
O10	6.384841001	0.251700790	-2.189925543
O11	8.489135053	1.685843517	-2.789635779
O12	5.546648397	1.394935816	3.161334759
H1	0.856918892	1.500300092	0.061216836
H2	3.492414301	-0.840286458	-0.933204420
H3	3.579443491	3.658432372	3.730576224
H4	4.474635245	5.391834040	-1.674035329
H5	7.275426160	1.998489653	0.444243005
H6	5.446353465	0.191443339	-1.902352136
H7	9.290852665	2.039713458	-2.376423176
H8	5.761702955	0.465321428	3.011363432

Model of the oligomer (O_{C3})

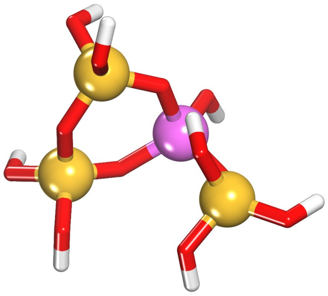

Si1	4.689674496	2.388067866	1.954583501
Si2	2.996344857	1.373060352	-0.264901859
O1	1.356527284	1.425369451	-0.107968025
O2	3.792389277	1.202883926	1.194312179
O3	3.385320298	-0.137706615	-0.886562295
O4	3.570544788	2.622561688	-1.093104676
O5	3.739056555	3.398199714	2.849753034
Al1	5.076181960	3.561904941	-0.849381488
Si3	7.258434544	1.541108840	-1.575936248
O6	5.566501837	3.191459113	0.851392270
O7	4.860101054	5.269525775	-1.001729124
O8	6.349212746	2.837240709	-1.856799660
O9	7.417790645	1.267815994	0.068328779
O10	6.543315529	0.069877036	-1.974276357
O11	8.697554349	1.575238808	-2.384903913
O12	5.634956195	1.457995586	2.949591336
H1	1.000355832	2.314477446	-0.261512063
H2	3.008336433	-0.327341565	-1.757806863
H3	3.727289245	4.302023381	2.495543251
H4	4.550667713	5.655570766	-1.830567372
H5	7.015952290	2.031895660	0.552505434
H6	6.331434745	-0.042502775	-2.912093766
H7	9.040946182	2.468983937	-2.539533331
H8	6.026522289	1.928711360	3.700555408
